# Circulating cell adhesion molecules in systemic sclerosis: a systematic review and meta-analysis

**DOI:** 10.3389/fimmu.2024.1438302

**Published:** 2024-08-21

**Authors:** Arduino A. Mangoni, Angelo Zinellu

**Affiliations:** ^1^ Discipline of Clinical Pharmacology, College of Medicine and Public Health, Flinders University, Adelaide, SA, Adelaide, Australia; ^2^ Department of Clinical Pharmacology, Flinders Medical Centre, Southern Adelaide Local Health Network, Adelaide, SA, Adelaide, Australia; ^3^ Department of Biomedical Sciences, University of Sassari, Sassari, Italy

**Keywords:** cell adhesion molecules, immunoglobulin-like cell adhesion molecules, selectins, integrins, cadherins, systemic sclerosis, biomarkers, endothelial activation

## Abstract

**Introduction:**

Patients with systemic sclerosis (SSc) have an increased risk of endothelial dysfunction, atherosclerosis, and cardiovascular events compared to the general population. Therefore, the availability of robust circulating biomarkers of endothelial dysfunction and atherogenesis may facilitate early recognition and management of cardiovascular risk in SSc. We sought to address this issue by conducting a systematic review and meta-analysis of studies investigating various types of circulating cell adhesion molecules involved in endothelial dysfunction and atherogenesis (i.e., immunoglobulin-like vascular cell, VCAM-1, intercellular, ICAM-1, platelet endothelial cell, PECAM-1, neural cell, NCAM, Down syndrome cell, DSCAM, and endothelial cell-selective, ESAM, adhesion molecules, E-, L-, and P-selectin, integrins, and cadherins) in SSc patients and healthy controls.

**Methods:**

We searched PubMed, Scopus, and Web of Science from inception to 1 May 2024. Risk of bias and certainty of evidence were assessed using validated tools.

**Results:**

In 43 eligible studies, compared to controls, patients with SSc had significantly higher plasma or serum concentrations of ICAM-1 (standard mean difference, SMD=1.16, 95% CI 0.88 to 1.44, p<0.001; moderate certainty), VCAM-1 (SMD=1.09, 95% CI 0.72 to 1.46, p<0.001; moderate certainty), PECAM-1 (SMD=1.65, 95% CI 0.33 to 2.98, p=0.014; very low certainty), E-selectin (SMD=1.17, 95% CI 0.72 to 1.62, p<0.001; moderate certainty), and P-selectin (SMD=1.10, 95% CI 0.31 to 1.90, p=0.007; low certainty). There were no significant between-group differences in L-selectin concentrations (SMD=-0.35, 95% CI -1.03 to 0.32, p=0.31; very low certainty), whereas minimal/no evidence was available for cadherins, NCAM, DSCAM, ESAM, or integrins. Overall, no significant associations were observed between the effect size and various patient and study characteristics in meta-regression and subgroup analyses.

**Discussion:**

The results of this systematic review and meta-analysis suggest that specific circulating cell adhesion molecules, i.e., ICAM-1, VCAM-1, PECAM-1, E-selectin, and P-selectin, can be helpful as biomarkers of endothelial dysfunction and atherogenesis in the assessment of cardiovascular risk in SSc patients.

**Systematic review registration:**

https://www.crd.york.ac.uk/prospero/, identifier CRD42024549710.

## Introduction

Systemic sclerosis (SSc), an autoimmune condition primarily affecting women, is characterized by vascular dysfunction and progressive fibrosis of the skin and internal organs (1, 2). The global incidence of SSc ranges between 8-56 new cases per million persons per year and the prevalence varies between 38-341 cases per million persons (3). The mortality in SSc patients is three- to four-fold higher than the general population due to cardiorespiratory complications, renal and gastrointestinal disease, cancer, and infections (4, 5). Increasing evidence also suggests that atherosclerosis is a critical additional component of the pathophysiology of SSc. This has led to a shift in the focus of basic and clinical research studies which have convincingly reported several pro-atherosclerotic arterial abnormalities, e.g., endothelial dysfunction, increased intima-media thickness and arterial stiffness, in SSc (6–10). Such alterations are similar to those observed in rheumatoid arthritis, another autoimmune condition associated with atherosclerosis and cardiovascular disease (11, 12). Epidemiological studies have also reported an increased risk of atherosclerotic cardiovascular events in SSc, particularly myocardial infarction and peripheral vascular disease (13, 14). In these studies, the prevalence and/or severity of hypertension, diabetes, and dyslipidemia in SSc patients was similar to that in control groups (9, 13). This suggests that conventional risk factors only partially account for the increased risk of atherosclerosis and cardiovascular disease in SSc. Therefore, a focus of current research is the identification of alternative, more robust biomarkers of atherosclerosis allowing early risk stratification and preventive treatment.

Functional and structural alterations of the endothelium, associated with the impaired synthesis of the critical endogenous messenger nitric oxide, represent the initial step in the pathogenesis of atherosclerosis (15). At a cellular and molecular level, these alterations involve the adhesion of leukocytes and lymphocytes to the endothelium (endothelial activation) and their consequent migration to the tunica intima, where they initiate a sequence of events leading to the formation of the atherosclerotic plaque (16, 17). The process of cellular adhesion to the endothelium is mediated by several molecules, e.g., the immunoglobulin-like vascular cell adhesion molecule-1 (VCAM-1), the intercellular vascular adhesion molecule-1 (ICAM-1), the platelet endothelial cell adhesion molecule-1 (PECAM-1), the neural cell adhesion molecule (NCAM), the Down syndrome cell adhesion molecule (DSCAM), and the endothelial cell-selective adhesion molecule (ESAM) (18–21). VCAM-1 is expressed in endothelial cells and macrophages and binds to integrin α_4_β_1_ (22, 23). ICAM-1 is upregulated during inflammation and binds to the leukocyte specific β_2_ integrins (24, 25). PECAM-1 is expressed in leukocytes, platelets, and endothelial cells, and exerts its effects through the translocation of integrin α_6_β_1_ (26). NCAM is expressed in the brain, skeletal muscle, and hematopoietic system. In addition to regulating cell adhesion, it modulates brain and kidney development and plays a pathophysiological role in cancer, schizophrenia, and other neurodegenerative disorders (27). DSCAM is primarily expressed in the brain and regulates neural development (28). ESAM is expressed mainly in endothelial cells and is critical in modulating angiogenesis, endothelial integrity, leukocyte adhesion and transmigration (29).

The immunoglobulin-like cell adhesion molecules can be measured in plasma or serum (21, 27, 28, 30, 31). Their concentrations, particularly VCAM-1, ICAM-1, and ESAM, have been shown to be associated with endothelial dysfunction, vascular damage, and increased risk of atherosclerotic cardiovascular disease (32–38). Other molecules facilitating cell adhesion to the endothelium include selectins, integrins, and cadherins (39, 40). The selectins include P-selectin, expressed in platelets and endothelial cells, L-selectin, expressed in leukocytes, and E-selectin, expressed in endothelial cells (41–43). L-selectin mediates lymphocyte rolling, whereas P-selectin and E-selectin influence the rolling of monocytes, neutrophils, and lymphocytes (44, 45). Similar to the immunoglobulin-like cell adhesion molecules, selectins, integrins, and cadherins can be measured in plasma or serum, and their concentrations have also been shown to be associated with an increased risk of atherosclerosis and cardiovascular disease (46–52).

To evaluate the possible role of cell adhesion molecules as biomarkers of endothelial activation, dysfunction, and atherosclerosis in SSc, we conducted a systematic review and meta-analysis of studies investigating their plasma or serum concentrations in SSc patients and healthy controls. Where possible, we investigated possible associations between the effect size of the between-group differences in cell adhesion molecules and pre-defined study and patient characteristics.

## Materials and methods

### Search strategy and study selection

We searched PubMed, Web of Science, and Scopus from inception to 21 July 2024 for relevant articles using the following terms: “systemic sclerosis” OR “scleroderma” AND “soluble cell adhesion molecules” OR “intercellular adhesion molecule” OR “ICAM” OR “sICAM” OR “ICAM” OR “vascular cell adhesion molecule” OR “VCAM” OR “sVCAM” OR “VCAM” OR “platelet endothelial cell adhesion molecule” OR “PECAM” OR “sPECAM” OR “PECAM” OR “Selectin” or “P-selectin” OR “sP-selectin” OR “L-selectin” OR “sL-selectin” OR “E-selectin” OR “sE-selectin” OR “ESAM” OR “sESAM” OR “endothelial cell-selective adhesion molecule” OR “NCAM” OR “sNCAM” OR “neural cell adhesion molecules” OR “DSCAM” OR “sDSCAM” OR “Down syndrome cell adhesion molecule” OR “integrins” OR “cadherin”.

Each abstract was screened by two independent investigators and reviewed as full text if considered relevant. Any disagreement between the investigators throughout the screening process was resolved by a third investigator. The inclusion criteria were: (a) the measurement of soluble ICAM-1, VCAM-1, PECAM-1, ESAM, NCAM, DSCAM, E-selectin, L-selectin, P-selectin, integrins, and cadherins in plasma or serum; (b) the comparison between SSc patients and healthy controls in original case-control research studies; (c) the inclusion of patients aged ≥18 years; and (d) the availability of the full text of the publication in English language. The exclusion criteria were: (a) the investigation of other autoimmune or autoinflammatory conditions; (b) case reports and review articles; and (c) the inclusion of children and/or adolescents. References of reviewed articles were also searched to identify additional studies.

Two investigators independently extracted the following data from each article: year of publication, first author, study country and continent, sample size, age, male to female ratio, SSc type (diffuse or localized), disease duration, concentrations of individual cell adhesion molecules, and biological matrix assessed (serum or plasma). The data were then manually transferred to separate custom extraction forms created using Microsoft Excel. Any discrepancy between the extraction forms was resolved by a third investigator.

The risk of bias was assessed using the Joanna Briggs Institute Critical Appraisal Checklist for analytical studies (53). The certainty of evidence was evaluated using the Grades of Recommendation, Assessment, Development, and Evaluation (GRADE) Working Group system (54). The study followed the Preferred Reporting Items for Systematic Reviews and Meta-Analyses (PRISMA) 2020 statement (**Supplementary Table 1**) (55). The protocol was registered in an international repository (PROSPERO registration number, CRD42024549710).

### Statistical analysis

Standardized mean differences (SMDs) and 95% confidence intervals (CIs) were calculated to generate forest plots and assess the differences in the concentrations of individual cell adhesion molecules between SSc patients and healthy controls. A p-value <0.05 was considered statistically significant. If required, data were extracted from graphs using the Graph Data Extractor software (San Diego, CA, USA). Means and standard deviations were calculated from medians and interquartile ranges or full ranges according to published methods (56). The heterogeneity of SMD across studies was evaluated using the Q-statistic (significance level at p<0.10) and classified as low (I^2^ ≤25%), moderate (25%< I^2^ <75%), or high (I^2^ ≥75%) (57, 58). Sensitivity analysis and assessment of publication bias were conducted according to established methods (59–62).

Univariate meta-regression and subgroup analyses were conducted to investigate associations between the effect size and the following parameters: year of publication, study continent, number of participants, age, male to female ratio, SSc type (diffuse or localized), mean disease duration, and biological matrix assessed (serum or plasma). Statistical analyses were performed using Stata 14 (Stata Corp., College Station, TX, USA).

## Results

### Study selection

**Figure 1:**
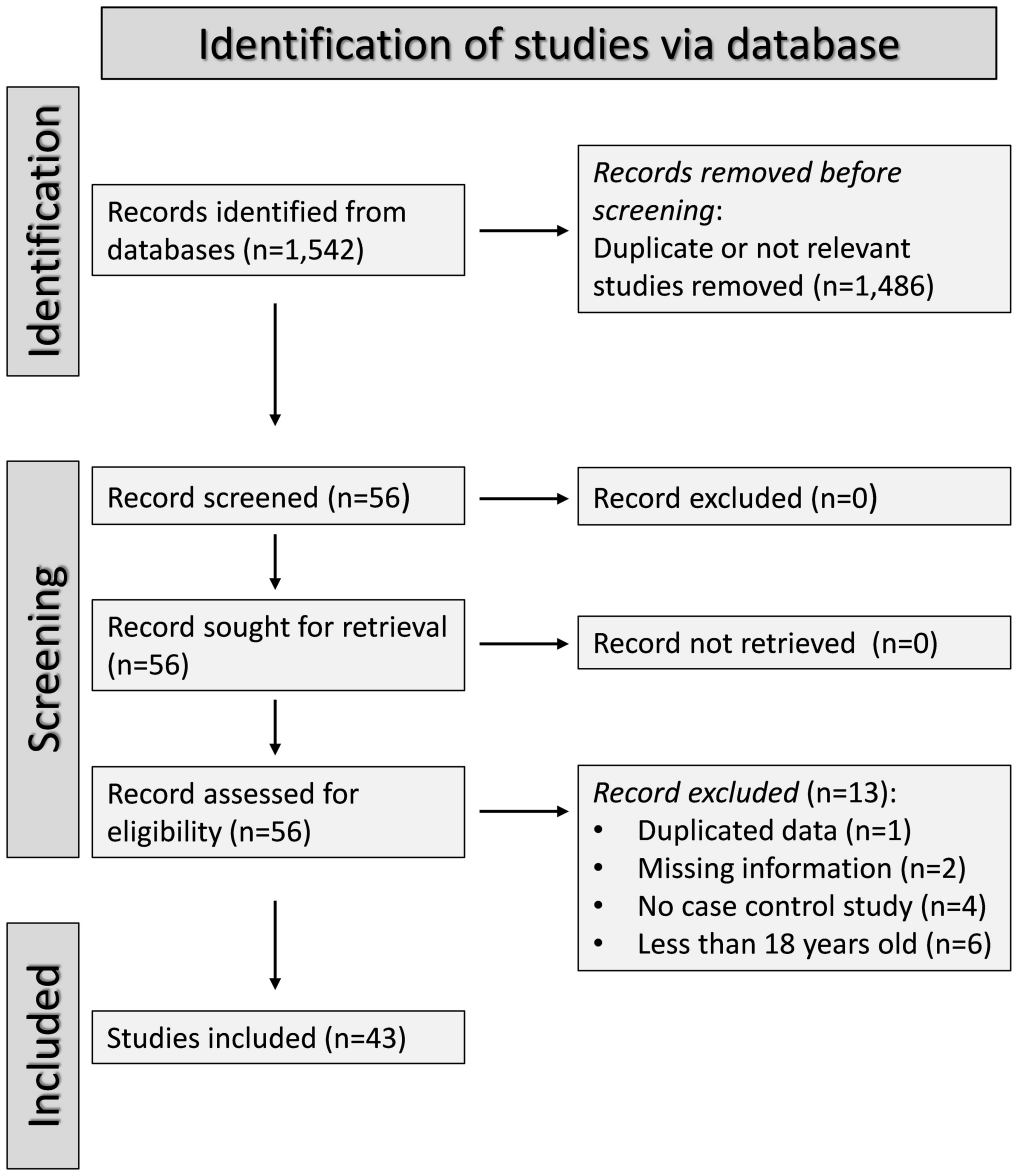
PRISMA 2020 flow diagram.

The flow chart of study selection is illustrated in **Figure 1**. After initially identifying 1,542 articles, 1,486 were excluded because they were either irrelevant or presented duplicate data. A full-text review of the remaining 56 articles led to the exclusion of one study because of duplicate data, two studies because of missing information, four studies because their design was not case-control, and six studies because they included patients under 18 years old. Therefore, 43 studies were included in the analysis (**Table 1**) (63–105). The risk of bias was low or moderate in all studies except one which was assessed as having high risk (84) (**Supplementary Table 2**). The initial level of the certainty of evidence was considered low because of the case-control design of the selected studies (level 2).

**Table 1:** Main characteristics and results of the studies included in the meta-analysis.

	Healthy controls					Patients with systemic sclerosis				
Study	n	Age (Years)	M/F	ICAM-1 VCAM-1 PECAM-1 (Mean ± SD)	E-Selectin L-Selectin P-Selectin VE-Cadherin (Mean ± SD)	n	Age (Years)	M/F	ICAM VCAM PECAM-1 ELAM-1 (Mean ± SD)	E-Selectin L-Selectin P-Selectin VE-Cadherin (Mean ± SD)
Carson et al., 1993, USA (63)	71	44.3	36/35	NR NR NR	1.2 ± 0.2 NR NR NR	69	47.8	16/53	NR NR NR	2.08 ± 0.5 NR NR NR
Sfikakis et al., 1993, USA (64)	22	31	13/9	373 ± 127 NR NR	NR NR NR NR	37	46	6/31	587 ± 207 NR NR	NR NR NR NR
Kiener et al., 1994, Austria (65)	82	47	38/44	312 ± 78 NR NR	NR NR NR NR	33	53	7/26	449 ± 135 NR NR	NR NR NR NR
Blann et al., 1995, UK (66)	80	45	39/41	319 ± 107 746 ± 265 NR	58 ± 20 NR NR NR	37	43	11/26	447 ± 212 1090 ± 649 NR	131 ± 83 NR NR NR
Gruschwitz et al., 1995, Germany (67)	36	NR	NR	150 ± 18 497 ± 43 NR	48 ± 19 NR 262 ± 85 NR	12	50.7	3/9	168 ± 26 652 ± 178 NR	46 ± 10 NR 258 ± 82 NR
Blann et al., 1996, UK (68)	42	48	21/21	NR NR NR	NR 1244 ± 269 NR NR	18	46	6/12	NR NR NR	NR 1190 ± 334 NR NR
Ihn et al., 1997, Japan (69)	20	NR	NR	192 ± 49 NR NR	NR NR NR NR	88	50.3	9/79	317 ± 170 NR NR	NR NR NR NR
Ihn et al., 1998, Japan (70)	20	matched	NR	NR 506.8 ± 126.3 NR	53.5 ± 14.6 NR NR NR	80	50	8/72	NR 786.6 ± 297.6 NR	83.7 ± 30.7 NR NR NR
Majewski et al., 1999, Poland (71)	25	NR	NR	207 ± 63 NR NR	NR NR NR NR	36	52.7	8/28	375 ± 123 NR NR	NR NR NR NR
Sfikakis et al., 1999, Greece (72)	40	matched	matched	NR NR NR	NR NR 245 ± 97 NR	25	49	6/19	NR NR NR	NR NR 332 ± 167 NR
Andersen et al., 2002, Sweden (73)	24	57.7	matched	NR 593 ± 121 NR	47.4 ± 12.8 NR NR NR	24	57.9	4/20	NR 768 ± 241 NR	60.3 ± 23.7 NR NR NR
Macko et al., 2002, USA (74)	22	matched	3/19	NR NR NR	37.8 ± 14.5 NR NR NR	45	51.5	3/42	NR NR NR	67.7 ± 50.3 NR NR NR
Blann et al., 2003, France (75)	38	54	5/33	NR NR NR	NR NR 44.7 ± 19.4 NR	67	54	6/61	NR NR NR	NR NR 144 ± 114 NR
Cerinic et al., 2003, Italy (76)	16	matched	matched	211 ± 48 472 ± 66 NR	25.7 ± 12.4 NR NR NR	29	45.3	2/27	353 ± 113 711 ± 247 NR	46 ± 17.8 NR NR NR
Zamzam et al., 2003, Egypt (77)	10	42	2/8	101.8 ± 13.67 NR NR	NR NR NR NR	20	48.0	5/15	163.75 ± 56.93 NR NR	NR NR NR NR
Allanore et al., 2004, France (78)	20	51	3/17	NR 482 ± 64 NR	NR NR NR NR	40	57	7/33	NR 705 ± 156 NR	NR NR NR NR
Ates et al., 2004, Turkey (79)	16	50.2	4/12	NR NR NR	24.9 ± 12.9 672 ± 140 292 ± 199 NR	30	47.8	3/27	NR NR NR	44.9 ± 22.3 552 ± 224 246 ± 163 NR
Kuryliszyn-Moskal et al., 2004, Poland (80)	30	matched	matched	NR 528.6 ± 172.9 NR	34.7 ± 12.1 NR NR NR	31	55.2	0/31	NR 682.6 ± 172.8 NR	47.3 ± 13.4 NR NR NR
Dovio et al., 2008, Italy (81)	60	55.5	12/48	NR 664 ± 80 NR	NR NR NR NR	60	54.8	12/48	NR 1032 ± 197 NR	NR NR NR NR
Hettema et al., 2008, The Netherlands (82)	32	50.9	3/29	NR 291 ± 88 NR	NR NR NR NR	49	55.4	8/41	NR 243 ± 94 NR	NR NR NR NR
Iannone et al., 2008, Italy (83)	25	46.7	NR	588 ± 48 261 ± 9 41 ± 2	NR NR 132 ± 12 NR	35	51.4	NR	3077 ± 903 301 ± 12 47.8 ± 3.4	NR NR 363 ± 58 NR
Nomura et al., 2009, Japan (84)	30	43	11/19	343 ± 30 492 ± 59 NR	42.3 ± 9.1 NR 112 ± 25 NR	42	48.4	7/35	335 ± 226 781 ± 73 NR	64.2 ± 11.2 NR 184 ± 25 NR
Minier et al., 2010, Hungary (85)	30	NR	NR	NR NR NR	31.6 ± 15 NR NR NR	131	55.9	12/119	NR NR NR	35.4 ± 15.4 NR NR NR
Olewicz-Gawlik et al., 2010, Poland (86)	30	47.1	2/28	NR NR NR	39.5 ± 20.8 NR 46.2 ± 20.7 NR	30	52.9	3/27	NR NR NR	50.2 ± 21.9 NR 132.8 ± 107.1 NR
Alzawawy et al., 2011, Egypt (87)	10	matched	3/7	NR 1214.33 ± 324.29 NR	NR NR NR NR	15	32.1	2/13	NR 1931.15 ± 593.52 NR	NR NR NR NR
Riccieri et al., 2011, Italy (88)	16	matched	matched	NR NR 12.4 ± 4.8	NR NR NR NR	65	53.9	2/63	NR NR 38.1 ± 28.7	NR NR NR NR
Dunne et al., 2012, Canada (89)	30	55.9	matched	NR NR NR	NR 906 ± 40 NR NR	30	55.3	6/24	NR NR NR	NR 885 ± 71 NR NR
Aydoğdu et al., 2013, Turkey (90)	20	49.3	1/19	NR NR NR	NR NR NR 2.73 ± 6.0	40	48.3	2/38	NR NR NR	NR NR NR 3.75 ± 5.8
Iversen et al., 2013, Denmark (91)	49	46	6/43	NR NR NR	27.4 ± 1.8 NR 34.13 NR	121	57	19/102	NR NR NR	41.9 ± 2 NR 43.2 ± 14.7 NR
Cossu et al. (a) 2016, Italy (92)	43	NR	NR	349.17 ± 222.75 619.95 ± 207.18 NR	22.6 ± 9 NR NR NR	95	57.4	NR	478.01 ± 318.65 583.07 ± 228.62 NR	25.1 ± 9.9 NR NR NR
Cossu et al. (b) 2016, Italy (92)	43	NR	NR	349.17 ± 222.75 619.95 ± 207.18 NR	22.6 ± 9 NR NR NR	86	59	NR	593.13 ± 405.34 669.65 ± 243.72 NR	28.4 ± 10.7 NR NR NR
Yalçınkaya et al., 2016, Turkey (93)	20	NR	NR	NR 3231 ± 1,435 NR	205 ± 78 NR 364 ± 137 NR	72	44.9	6/66	NR 3945 ± 1754 NR	269 ± 106 NR 287 ± 86 NR
Delle Sedie et al., 2018, Italy (94)	31	53.5	6/25	21.57 ± 5.07 11.05 ± 3.84 NR	NR NR NR NR	41	54	1/40	31.33 ± 16.00 14.84 ± 6.28 NR	NR NR NR NR
Thakkar et al., 2018, Australia (95)	34	51.2	NR	201.8 ± 57.2 1125.6 ± 273.4 NR	NR NR NR NR	64	52.6	6/58	297.4 ± 134 1432.7 ± 427.4 NR	NR NR NR NR
Wodok-Wieczorek et al., 2018, Poland (96)	41	46.3	13/28	NR NR NR	25.3 ± 12.9 NR NR NR	42	49	7/35	NR NR NR	43.62 ± 21.7 NR NR NR
Hegazy et al., 2019, Egypt (97)	60	41.58	20/40	158.49 ± 43.31 NR NR	NR NR NR NR	30	47.31	7/23	437.62 ± 175.52 NR NR	NR NR NR NR
Pacholczak-Madej et al., 2020, Poland (98)	36	56.3	11/25	NR 818 ± 160 NR	NR NR NR NR	42	59.2	7/35	NR 858 ± 256 NR	NR NR NR NR
Al-Omary Obadeh et al., 2021, Ukraine (99)	35	39.5	13/22	NR 339 ± 184 NR	NR NR NR NR	78	43.2	27/51	NR 685 ± 454 NR	NR NR NR NR
Kuszmiersz et al., 2021, Poland (100)	56	50	9/48	NR 816 ± 61 NR	NR NR NR NR	67	57	16/51	NR 852 ± 76 NR	NR NR NR NR
Stern et al., 2021, UK (101)	12	34	NR	14287 ± 6055 22427 ± 14297 NR	NR NR NR NR	40	57	NR	25449 ± 11316 23291 ± 11645 NR	NR NR NR NR
Brezovec et al., 2022, Slovenia (102)	36	55	5/31	NR NR NR	NR 1128 ± 99 NR NR	38	57	7/31	NR NR NR	NR 1190 ± 334 NR NR
Colic et al., 2022, Serbia (103)	46	51.3	5/41	24.3 ± 4.6 30.8 ± 8.8 NR	4.1 ± 1.7 NR NR NR	58	54.3	7/51	29.5 ± 7.8 37.3 ± 11.3 NR	5.2 ± 1.4 NR NR NR
Jee et al., 2023, Australia (104)	30	36.8	5/25	370 ± 172 0.93 ± 0.23 NR	24.1 ± 10.4 NR NR NR	179	57.9	31/148	657 ± 324 1.27 ± 0.62 NR	38.4 ± 18 NR NR NR
Corrado et al., 2024, Italy (105)	37	57.86	4/33	NR 391.27 ± 102.54 NR	NR NR NR NR	57	58.91	5/52	NR 738.15 ± 123 NR	NR NR NR NR

ELAM-1, Endothelial leucocyte adhesion molecule-1; ICAM-1, intercellular adhesion molecule-1; M/F, male to female ratio; NR, not reported; PECAM-1, Platelet/endothelial cell adhesion molecule-1; VCAM-1, vascular cell adhesion molecule-1.

### ICAM-1

Seventeen studies, including 18 group comparisons, reported ICAM-1 concentrations in 962 SSc patients (mean age 53 years, 85% females) and 645 healthy controls (mean age 45 years, 65% females) (64–67, 69, 71, 76, 77, 83, 84, 92, 94, 95, 97, 101, 103, 104) (**Table 1**). Ten studies were conducted in Europe (65–67, 71, 76, 83, 92, 94, 101, 103) and the remaining seven in other geographical areas (64, 69, 77, 84, 95, 97, 104). Measurements were conducted in serum in 14 studies (65–67, 69, 71, 77, 83, 92, 94, 95, 97, 101, 103, 104), plasma in two (76, 84), and both plasma and serum in the remaining one (64). Ten studies reported disease duration, ranging between 1.7 and 14.6 years (65, 67, 69, 77, 83, 92, 94, 95, 97, 104) and ten whether the disease was diffuse or localized (64, 65, 69, 71, 76, 92, 94, 95, 101, 103).

The risk of bias was considered low or moderate in all studies except one, which was assessed as having high risk (84) (**Supplementary Table 2**).

**Figure 2:**
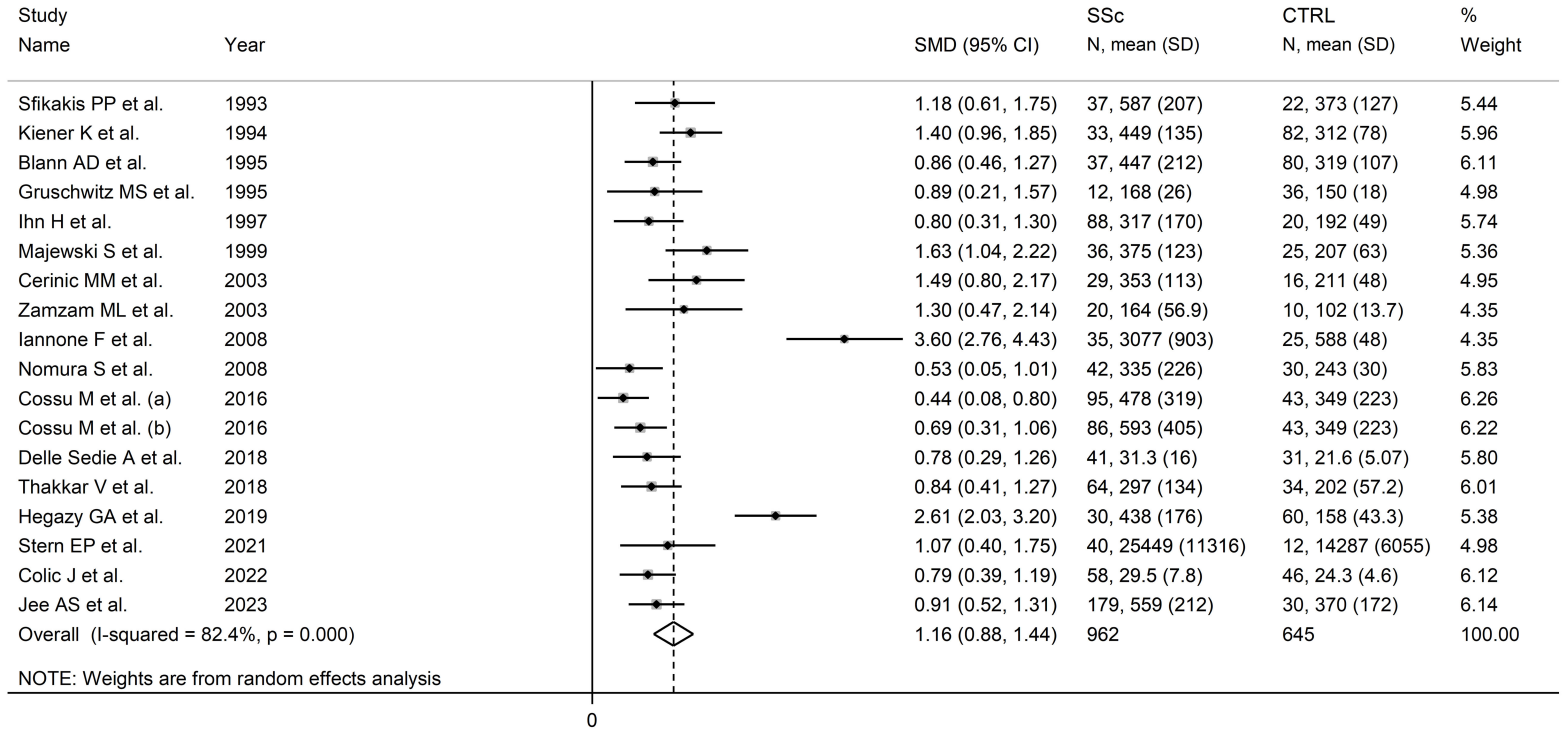
Forest plot of studies investigating ICAM-1 concentrations in SSc patients and controls.

Pooled analyses showed that ICAM-1 concentrations were significantly higher in SSc patients than controls (SMD=1.16, 95% CI 0.88 to 1.44, p<0.001; I^2^ = 82.4%, p<0.001; **Figure 2**). Sensitivity analysis showed stability of the results with pooled SMD values ranging between 1.04 and 0.84; **Supplementary Figure 1**).

There was significant publication bias (Begg’s test, p=0.002; Egger’s test, p=0.003). The “trim-and-fill” method was consequently used to address and correct this bias (62). This method operates under the assumption that the results of some studies, often those with null or negative findings, might be missing, leading to an asymmetric distribution in the funnel plot. Consequently, it estimates the number of such missing studies and adds them to the funnel plot to create a symmetrical distribution. This adjustment helps in evaluating the impact of publication bias on the overall results. The meta-analysis is then recalculated to include these additional studies. In this case, the “trim-and-fill” method identified seven missing studies to be added to the left side of the funnel plot to ensure symmetry (**Supplementary Figure 2**). This adjustment led to an attenuation of the resulting SMD however the effect size remained significant (SMD=0.76, 95% CI 0.44 to 0.90, p<0.001). This suggests that while there was evidence of publication bias, the overall effect size of ICAM-1 concentrations remained robust.

Univariate meta-regression analysis did not show any significant associations between the effect size and age (t=-0.26, p=0.82), male to female ratio (t=0.25, p=0.81), year of publication (t=-0.33, p=075), sample size (t=-1.30, p=0.21), and SSc duration (t=0.05, p=0.96). In sub-group analyses, there were non-significant differences (p=0.92) in pooled SMD between studies conducted in Europe (SMD=1.17, 95% CI 0.80 to 0.55, p<0.001; I^2^ = 83.6%, p<0.001) and other geographical areas (SMD=1.15, 95% CI 0.68 to 1.62, p<0.001; I2 = 82.8%, p<0.001). There were non-significant differences (p=0.72) in pooled SMD between studies measuring serum (SMD=1.19, 95% CI 0.87 to 1.51, p<0.001; I^2^ = 84.5%, p<0.001) and plasma (SMD=0.97, 95% CI 0.04 to 1.91, p=0.041; I^2^ = 80.2%, p=0.025). Similarly, there were non-significant differences (p=0.70) in pooled SMD between studies with a diffuse/localized disease patient ratio <1 (SMD=1.30, 95% CI 0.81 to 1.78, p<0.001; I^2^ = 82.8%, p<0.001) and >1 (SMD=1.14, 95% CI 0.78 to 1.50, p<0.001; I^2^ = 36.0%, p=0.21), with a reduced between-study variance in the >1 subgroup.

The overall level of certainty was upgraded to moderate (level 3) after considering the low-moderate risk of bias in most studies (no change), the high but partially explainable heterogeneity (no change), the lack of indirectness (no change), the large effect size (SMD=1.16; upgrade one level) (106), and the presence of publication bias which was addressed with the “trim-and-fill” method (no change).

### VCAM-1

Twenty-three studies, including 24 group comparisons, reported VCAM-1 concentrations in 1,413 SSc patients (mean age 54 years, 84% females) and 806 healthy controls (mean age 49 years, 75% females) (66, 67, 70, 73, 76, 78, 80–84, 87, 92–95, 98–101, 103–105) (**Table 1**). Seventeen were conducted in Europe (66, 67, 73, 76, 78, 80–83, 92, 94, 98–101, 103, 105) and the remaining six in other geographical areas (70, 84, 87, 93, 95, 104). Measurements were conducted in serum in 18 studies (66, 67, 70, 78, 80–83, 87, 92–95, 98, 101, 103–105) and plasma in five (73, 76, 84, 99, 100). Disease duration, reported in 15 studies, ranged between 2.6 and 14.6 years (66, 70, 78, 80, 81, 83, 87, 92–95, 98, 100, 104, 105). Fourteen studies reported whether SSc was localized or diffuse (70, 73, 76, 80, 87, 92–95, 98, 100, 101, 103, 105).

The risk of bias was considered low or moderate in all studies except one, which was assessed as having high risk (84) (**Supplementary Table 2**).

**Figure 3:**
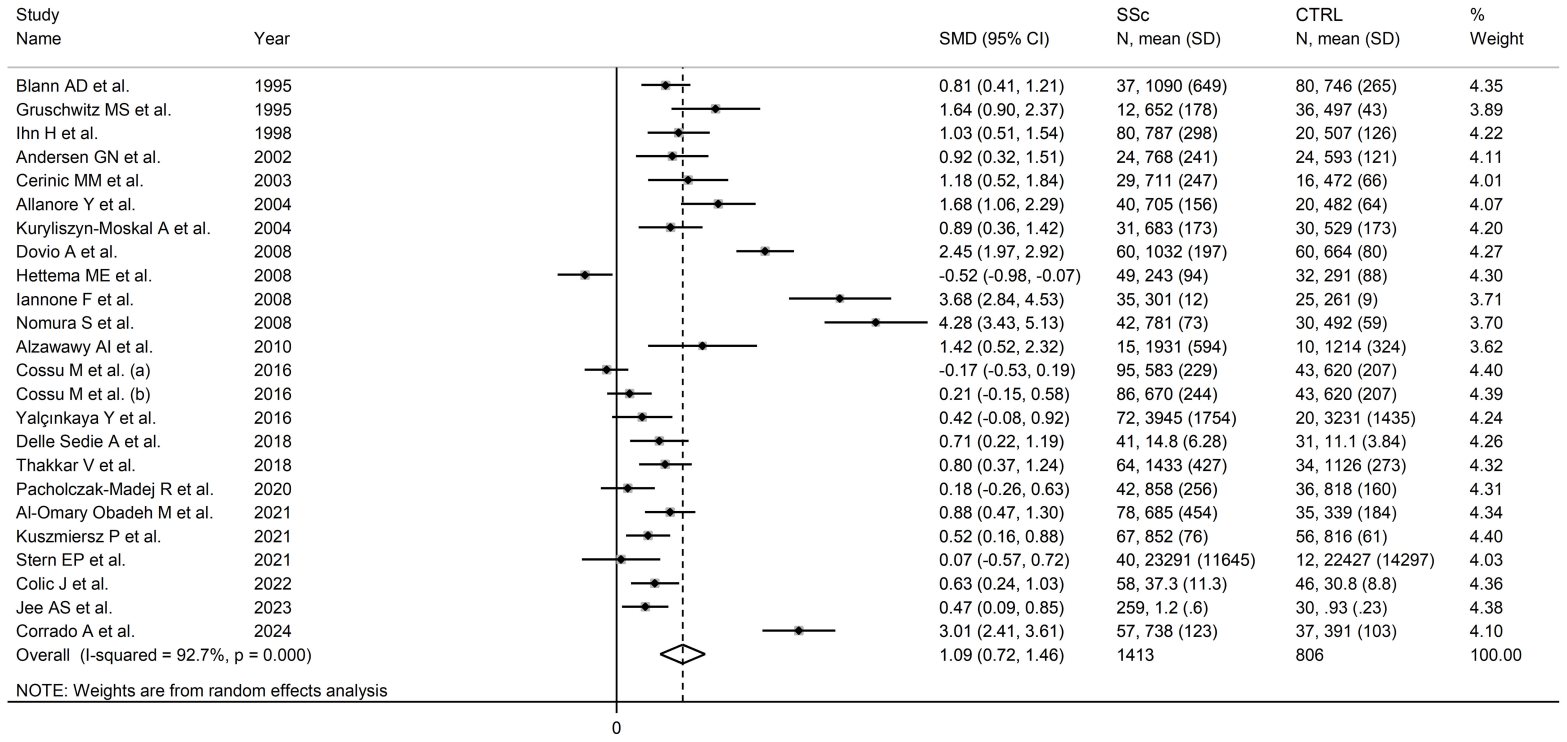
Forest plot of studies investigating VCAM-1 concentrations in SSc patients and controls.

Pooled analyses showed that VCAM-1 concentrations were significantly higher in SSc than controls (SMD=1.09, 95% CI 0.72 to 1.46, p<0.001; I^2^ = 92.7%, p<0.001; **Figure 3**). The results were stable in sensitivity analysis, with pooled SMD values ranging between 0.96 and 1.16 (**Supplementary Figure 3**).

There was significant publication bias (Begg’s test, p<0.001; Egger’s test, p=0.001). The “trim-and-fill” method identified seven missing studies to be added to the left side of the funnel plot to ensure symmetry (**Supplementary Figure 4**). The resulting SMD was attenuated but remained significant (SMD=0.51, 95% CI 0.08 to 0.94, p=0.021)

No significant associations were found between the effect size and age (t=-0.96, p=0.35), male to female ratio (t=-1.59, p=0.13), publication year (t=-1.07, p=0.30), sample size (t=-1.13, p=0.27), or SSc duration (t=1.29, p=0.22) in univariate meta-regression analysis. In sub-group analysis, there were non-significant differences (p=0.55) in pooled SMD between studies conducted in Europe (SMD=1.01, 95% CI 0.58 to 1.44, p<0.001; I^2^ = 93.0%, p<0.001) and other geographical areas (SMD=1.34, 95% CI 0.52 to 2.17, p=0.001; I^2^ = 92.9%, p<0.001). Non-significant differences (p=0.40) in pooled SMD were also observed between studies measuring serum (SMD=0.99, 95% CI 0.57 to 1.40, p<0.001; I^2^ = 92.7%, p<0.001) and plasma (SMD=1.50, 95% CI 0.55 to 2.45, p=0.002; I^2^ = 93.8%, p<0.001). Similarly, non-significant differences (p=0.56) in pooled SMD were observed between studies with a diffuse/localized disease patient ratio <1 (SMD=0.92, 95% CI 0.43 to 1.41, p<0.001; I^2^ = 88.4%, p<0.001) and >1 (SMD=0.62, 95% CI 0.27 to 0.97, p=0.001; I^2^ = 59.0%, p=0.045), with lower between-study variance in the >1 subgroup.

The overall level of certainty was upgraded to moderate (level 3) after considering the low-moderate risk of bias in most studies (no change), the high but partially explainable heterogeneity (no change), the lack of indirectness (no change), the large effect size (SMD=1.09; upgrade one level) (106), and the presence of publication bias which was addressed with the “trim-and-fill” method (no change).

### PECAM-1

Two European studies reported PECAM-1 concentrations in 100 SSc patients and 41 healthy controls (83, 88) (**Table 1**). Measurements were conducted in serum in one study (83) and plasma in the other (88). The risk of bias was low in one study (83) and moderate in the other (88) (**Supplementary Table 2**).

**Figure 4:**
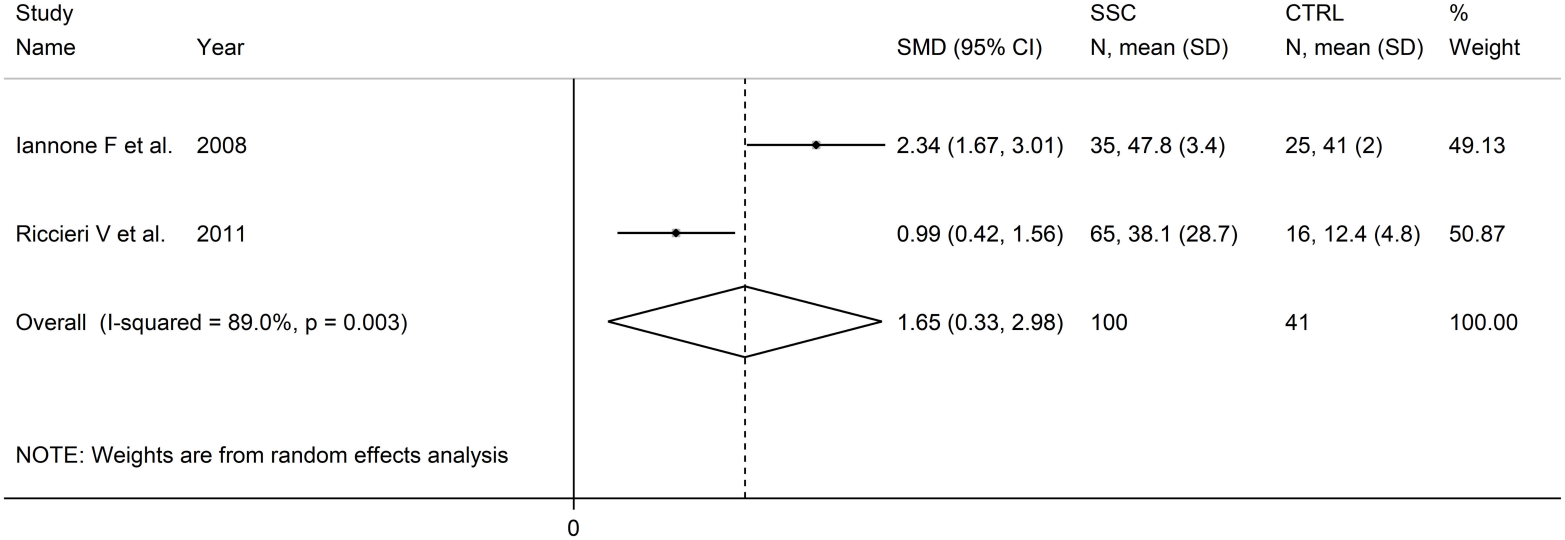
Forest plot of studies investigating PECAM-1 concentrations in SSc patients and controls.

Pooled analyses showed that PECAM-1 concentrations were significantly higher in SSc patients compared to controls (SMD=1.65, 95% CI 0.33 to 2.98, p=0.014; I^2^ = 89.0%, p=0.003; **Figure 4**). Assessment of the risk of bias, meta-regression, and subgroup analyses could not be performed because of the small number of studies. The overall level of evidence was downgraded to very low (level 1) because of the high and unexplained heterogeneity and the lack of assessment of publication bias.

### E-selectin

Eighteen studies, including 19 group comparators, assessed E-selectin concentrations in 1,293 SSc patients (mean age 54 years, 86% females) and 677 healthy controls (mean age 48 years, 70% females) (63, 66, 67, 70, 73, 74, 76, 79, 80, 84–86, 91–93, 96, 103, 104) (**Table 1**). Eleven studies were conducted in Europe (66, 67, 73, 76, 80, 85, 86, 91, 92, 96, 103) and seven in other continents (63, 70, 74, 79, 84, 93, 104). Thirteen studies investigated serum (63, 66, 67, 70, 79, 80, 85, 86, 92, 93, 96, 103, 104) and five plasma (73, 74, 76, 84, 91). Disease duration, reported in 12 studies, ranged between 2.6 and 13.6 years (67, 70, 73, 74, 79, 80, 85, 86, 91–93, 104). Disease type (diffuse or localized) was reported in ten studies (70, 73, 74, 76, 80, 86, 91–93, 103).

The risk of bias was considered low or moderate in all studies, except one which was assessed as having high risk (84) (**Supplementary Table 2**).

**Figure 5:**
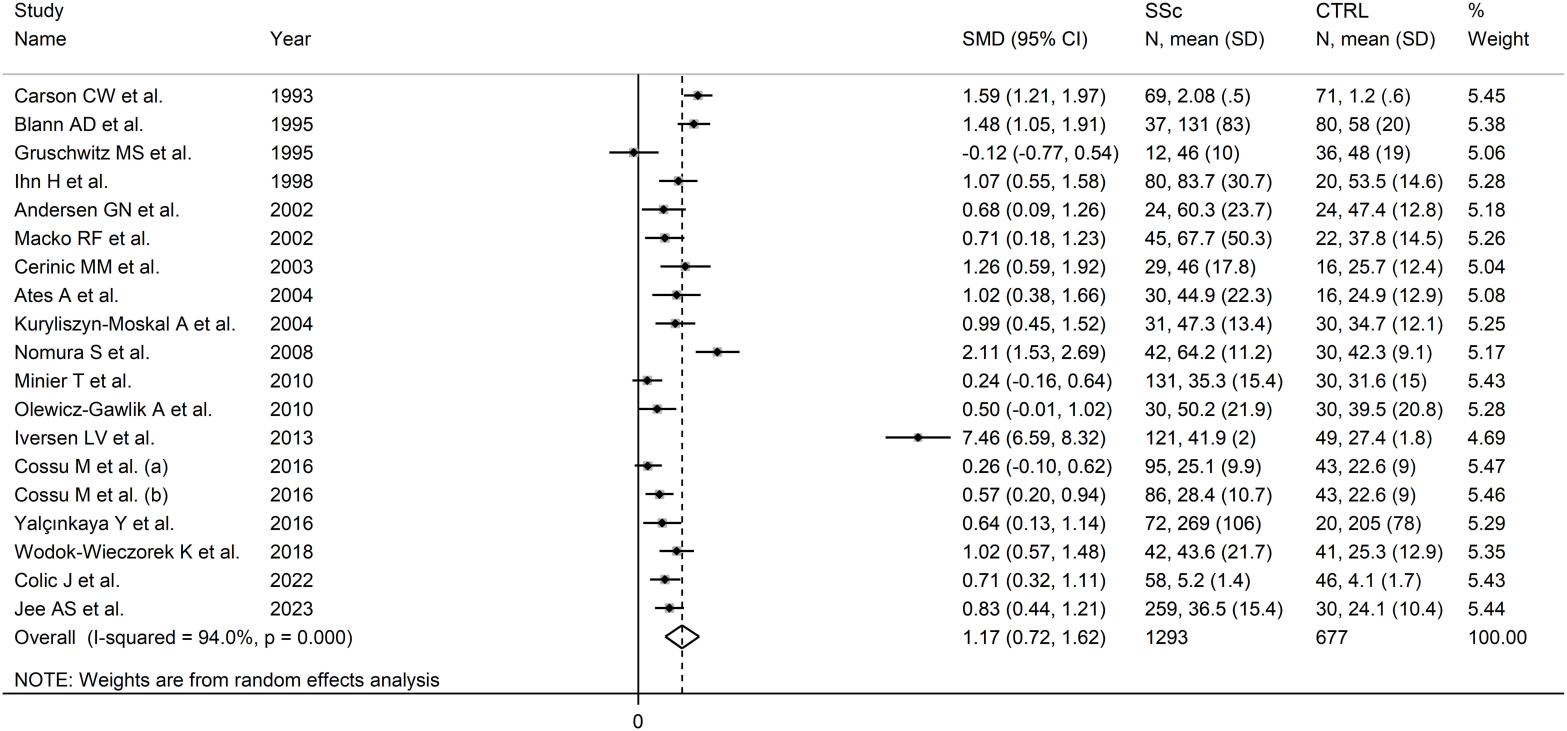
Forest plot of studies investigating E-selectin concentrations in SSc patients and controls.

Pooled analysis showed that SSc patients had significantly higher E-selectin concentrations when compared to controls (SMD=1.17, 95% CI 0.72 to 1.62, p<0.001; I^2^ = 94.0%, p<0.001; **Figure 5**). Sensitivity analysis showed that the pooled SMD values remained stable, ranging between 0.86 and 1.24 (**Supplementary Figure 5**), although one study, by Iversen et al, significantly influenced the effect size (91). This study also had a distortive effect on the funnel plot (**Supplementary Figure 6**). Its removal led to an attenuation of the effect size, which, however, remained significant (SMD=0.86, 95% CI 0.62 to 1.10, p<0.001), with lower between-study variance (I^2^ = 77.9, p<0.001).

No publication bias was observed after removing the study by Iversen et al. (91) (Begg’s test, p=0.26; Egger’s test, p=0.53). The “trim-and-fill” did not identify any missing studies to be added to the funnel plot to ensure symmetry (**Supplementary Figure 7**).

Meta-regression analysis did not show any significant associations between the effect size and age (t=0.86, p=0.41), male to female ratio (t=0.70, p=0.50), year of publication (t=0.17, p=0.87), sample size (t=0.96, p=0.35), or SSc duration (t=1.15, p=0.27). In sub-group analysis, there were non-significant differences (p=0.91) in pooled SMD between studies conducted in Europe (SMD=1.21, 95% CI 0.53 to 1.89, p<0.001; I^2^ = 95.9%, p<0.001) and other continents (SMD=1.13, 95% CI 0.75 to 1.51, p<0.001; I^2^= 76%, p=0.003), with a lower between-study variance in the non-European subgroup. By contrast, a significant difference (p=0.044) in pooled SMD was observed between studies investigating serum (SMD=0.78, 95% CI 0.53 to 1.03, p<0.001; I^2^ = 76.7%, p<0.001) and plasma (SMD=2.42, 95% CI 0.45 to 4.39, p=0.016; I^2^ = 94.1%, p<0.001), with a lower heterogeneity in the serum subgroup. Finally, non-significant differences (p=0.52) in pooled SMD were observed between studies with a diffuse/localized disease patient ratio <1 (SMD=1.73, 95% CI 0.53 to 2.93, p=0.005; I^2^ = 97.3%, p<0.001) and >1 (SMD=0.73, 95% CI 0.40 to 1.07, p<0.001; I^2^ = 21.9%, p=0.28), with reduced between-study variance in the >1 subgroup.

The overall level of certainty was upgraded to moderate (level 3) after considering the low-moderate risk of bias in most studies (no change), the high but partially explainable heterogeneity (no change), the lack of indirectness (no change), the large effect size (SMD=1.17; upgrade one level) (106), and the absence of publication bias (no change).

### L-selectin

Five studies assessed L-selectin concentrations in 141 SSc patients (mean age 52 years, 80% females) and 164 healthy controls (mean age 51 years, 68% females) (68, 72, 79, 89, 102) (**Table 1**). Four studies were conducted in Europe (68, 72, 89, 102) and one in Asia (79). Measurements were conducted in serum except one study which investigated plasma (89).

All studies had a low or moderate risk of bias (**Supplementary Table 2**).

**Figure 6:**
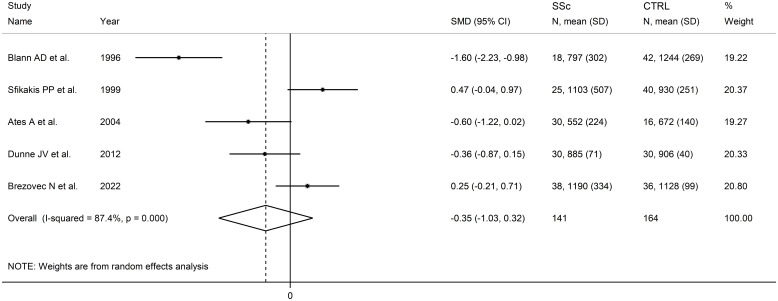
Forest plot of studies investigating L-selectin concentrations in SSc patients and controls.

Pooled analyses showed that L-selectin concentrations were non-significantly different between SSc patients and controls (SMD=-0.35, 95% CI -1.03 to 0.32, p=0.31; I^2^ = 87.4%, p<0.001; **Figure 6**). The pooled SMD values were stable in sensitivity analysis, ranging between -0.56 and -0.29 (**Supplementary Figure 8**).

Assessment of publication bias, meta-regression and sub-group and analysis could not be performed because of the small number of studies.

The overall level of evidence was downgraded to very low (level 1) because of the high and unexplained heterogeneity and the lack of assessment of publication bias.

### P-selectin

Nine studies assessed P-selectin concentrations in 434 SSc patients (mean age 52 years, 87% females) and 284 healthy controls (mean age 48 years, 82% females) (67, 72, 75, 79, 83, 84, 86, 91, 93) (**Table 1**). Six studies were conducted in Europe (67, 72, 75, 83, 86, 91) and three in Asia (79, 84, 93). Measurement was performed in serum in six studies (67, 72, 79, 83, 86, 93) and plasma in the remaining three (75, 84, 91).

The risk of bias was considered low or moderate in all studies except one which was assessed as having high risk (84) (**Supplementary Table 2**).

**Figure 7:**
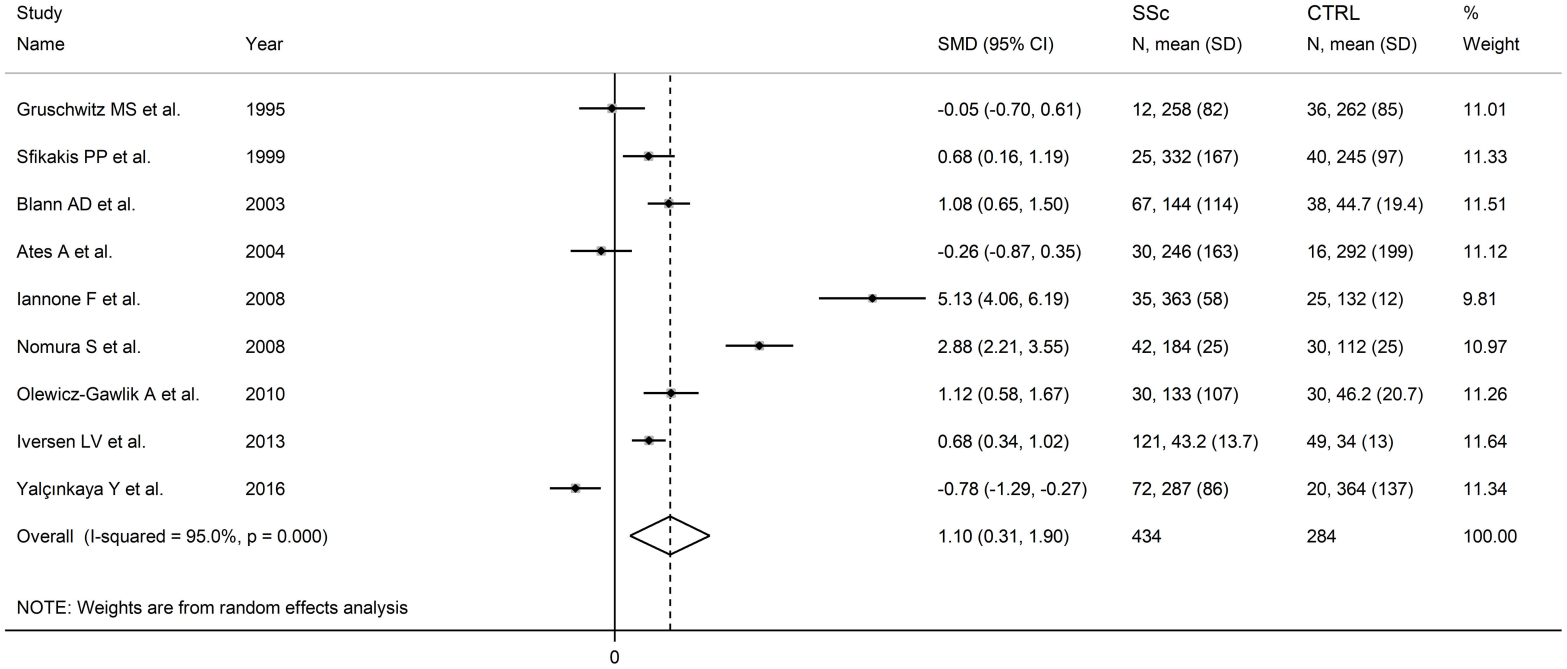
Forest plot of studies investigating P-selectin concentrations in SSc patients and controls.

Pooled analyses showed that SSc patients had significantly higher P-selectin concentrations when compared to controls (SMD=1.10, 95% CI 0.31 to 1.90, p=0.007; I^2^ = 95.0%, p<0.001; **Figure 7**). The results were stable in sensitivity analysis, with pooled SMD values ranging between 0.66 and 1.34 (**Supplementary Figure 9**).

Assessment of publication bias meta-regression analysis could not be conducted given the relatively small number of studies. In sub-group analysis, the pooled SMD was significantly higher in studies conducted in Europe (SMD=1.33, 95% CI 0.52 to 2.13, p=0.001; I^2^ = 93.1%, p<0.001) but not in other continents (SMD=0.61, 95% CI -1.53 to 2.74, p=0.58; I^2^ = 97.4%, p<0.001). The pooled SMD was also significantly higher in studies assessing plasma (SMD=1.51, 95% CI 0.44 to 2.58, p=0.006; I^2^= 93.9%, p<0.001) but not serum (SMD=0.91, 95% CI -0.25 to 2.07, p=0.13; I^2^ = 95.5%, p<0.001). Finally, the pooled SMD was significantly higher in studies with diffuse/localized disease patient ratio >1 (SMD=0.86, 95% CI 0.47 to 1.25, p<0.001; I^2^ = 51.5%, p=0.151) but not <1 (SMD=0.34, 95% CI -0.79 to 1.47, p=0.56; I^2^ = 92.9%, p<0.001), with lower heterogeneity in the >1 subgroup.

The overall level of certainty remained low (level 2) after considering the low-moderate risk of bias in most studies (no change), the high but partially explainable heterogeneity (no change), the lack of indirectness (no change), the large effect size (SMD=1.10; upgrade one level) (106), and the lack of assessment of publication bias (downgrade one level).

### Cadherins

One study with a low risk of bias (**Supplementary Table 2**) conducted in Turkey assessed vascular endothelium (VE)-cadherin in serum in 20 SSc patients and 40 healthy controls. Significantly higher VE-cadherin concentrations were observed in SSc patients (3.75 ± 5.8 vs 2.73 ± 6.0 pg/mL, p=0.016) (90) (**Table 1**).

### NCAM, DSCAM, ESAM, and integrins

No studies investigating these cell adhesion molecules in SSc and healthy controls were identified.

## Discussion

The results of this systematic review and meta-analysis have highlighted the presence of significant elevations in the concentrations of specific cell adhesion molecules, markers of endothelial activation, dysfunction, and atherogenesis, in patients with SSc. Such elevations were particularly evident in studies investigating ICAM-1, VCAM-1, PECAM-1, E-selectin, and P-selectin. The results were stable in sensitivity analysis, and the effect size of the observed between-group differences was generally not associated with individual study and patient characteristics. In particular, the lack of significant associations with SSc duration supports the proposition that elevations in cell adhesion molecules are already present in SSc patients with early disease, further supporting their potential clinical utility in assessing atherosclerotic burden. By contrast, no between-group differences were observed with L-selectin and minimal/no evidence was available for NCAM, DSCAM, ESAM, cadherins, and integrins.

Epidemiological studies have highlighted the increased risk of atherosclerotic cardiovascular events in SSc. The largest, conducted in Denmark, used data from administrative sources between 1995 and 2015 to identify patients with SSc and age- and sex-matched controls in a 1:5 ratio (14). Over a follow-up period of 8.9 years, SSc patients (n=2778) had a significantly increased risk of myocardial infarction (hazard ratio, HR=2.08, 95% CI 1.65 to 2.64), ischemic stroke (HR=1.28, 95% CI 1.04 to 1.58), and peripheral vascular disease (HR=5.73, 95% CI 4.63 to 7.09). These associations were maintained, except for ischemic stroke (HR=1.13, 95% CI 0.90 to 1.42), after adjusting for co-morbidities and medications. Our analyses suggest that measuring specific cell adhesion molecules might be helpful in evaluating atherosclerotic burden, stratifying cardiovascular risk, and facilitating the initiation of preventive strategies in SSc patients. Pending further research, assessing cell adhesion molecules may be particularly useful to demonstrate early endothelial dysfunction in absence of overt clinical evidence of vascular damage and atherosclerosis. However, an important issue to be addressed in further studies is whether such alterations in cell adhesion molecules might reflect endothelial dysfunction not only in the microcirculation, a vascular territory primarily affected in SSc (107, 108), but also in middle-size and large arteries, typically affected by the atherosclerotic process (7, 109–111).

Several studies have reported significant alterations in surrogate markers of endothelial function and arterial stiffness and an increased atherosclerotic burden in SSc (6–10). Endothelial cell injury, the main promoter of these alterations, stimulates adhesion and transmigration of leukocytes and monocytes into the tunica media of the arterial wall, initiating a sequence of events leading to the formation of the atherosclerotic plaque (8). A number of factors have been proposed as triggers of endothelial cell injury in SSc, including viruses (e.g., cytomegalovirus and Epstein Barr virus) (112, 113), cytotoxic CD4+ and CD8+ T-cells (114), autoantibodies against endothelial cells (115), and oxidative stress (116). However, further research is warranted to determine their role *in vivo*.

The capacity to restore endothelial function in SSc pharmacologically has been reported with dihydropyridine calcium channel blockers, statins, nitrate, endothelin-1 receptor antagonists, phosphodiesterase-5 inhibitors, soluble guanylate cyclase activators, prostacyclins, and cyclophosphamide (8),. Notably, dihydropyridine calcium channel blockers (117), statins (118, 119), endothelin-1 receptor antagonists (120), phosphodiesterase-5 inhibitors (121), soluble guanylate cyclase activators (122), and prostacyclins (123) have also been shown to downregulate several cell adhesion molecules *in vitro* and *in vivo*. Furthermore, systematic reviews and meta-analyses on the effects of statins on cell adhesion molecules in other patient populations have shown an effect size (SMD) on VCAM-1 and ICAM-1 between -0.28 and -0.75 and on E-, L-, and P-selectin between -0.39 and -0.73 (118, 119). The magnitude of these effects suggests that statins may be effective in reducing the circulating concentrations of cell adhesion molecules in SSc. Regardless, further research should investigate whether the measurement of cell adhesion molecules in SSc patients receiving these therapies may reflect a state of improved endothelial function and reduced atherosclerotic burden.

Strengths of our study include the comprehensive assessment of a wide range of cell adhesion molecules, the robust evaluation of the certainty of evidence for each cell adhesion molecule investigated, the evaluation of specific study and patient characteristics associated with the effect size by means of meta-regression and subgroup analysis, and the generalizability of our findings to different geographical areas although most studies were conducted in European countries. One important limitation is represented by the generally high heterogeneity observed which, however, could be partially explained for some cell adhesion molecules (ICAM-1 and VCAM-1: diffuse/localized disease patient ratio; E-selectin: study continent, biological matrix assessed, and diffuse/localized disease patient ratio; P-selectin: diffuse/localized disease patient ratio).

In conclusion, our study has shown significant elevations of specific cell adhesion molecules, i.e., ICAM-1, VCAM-1, PECAM-1, E-selectin, and P-selectin in SSc, which reflects a state of endothelial activation, dysfunction, and atherogenesis in this patient group. Pending the results of further prospective studies in patients with subclinical and overt atherosclerosis, which also investigate the microcirculation and the effect of specific therapies, cell adhesion molecules may assist in cardiovascular risk stratification in SSc.

## Data Availability

The raw data supporting the conclusions of this article will be made available by the authors, without undue reservation.

## Supplementary material

Supplementary Figure 1 Sensitivity analysis of the association between ICAM-1 concentrations and SSc.

Supplementary Figure 2 Funnel plot of studies investigating the association between ICAM-1 concentrations and SSc after “trimming-and-filling”. Dummy studies and genuine studies are represented by enclosed circles and free circles, respectively.

Supplementary Figure 3 Sensitivity analysis of the association between VCAM-1 concentrations and SSc.

Supplementary Figure 4 Funnel plot of studies investigating the association between VCAM-1 concentrations and SSc after “trimming-and-filling”. Dummy studies and genuine studies are represented by enclosed circles and free circles, respectively.

Supplementary Figure 5 Sensitivity analysis of the association between E-selectin concentrations and SSc.

Supplementary Figure 6 Funnel plot of studies investigating the association between E-selectin concentrations and SSc.

Supplementary Figure 7 Funnel plot of studies investigating the association between E-selectin concentrations and SSc after “trimming-and-filling”. Dummy studies and genuine studies are represented by enclosed circles and free circles, respectively.

Supplementary Figure 8 Sensitivity analysis of the association between L-selectin concentrations and SSc.

Supplementary Figure 9 Sensitivity analysis of the association between P-selectin concentrations and SSc.

## References

[1] Allanore Y Simms R Distler O Trojanowska M Pope J Denton CP . Systemic sclerosis. Nat Rev Dis Primers. (2015) 1:15002. doi: 10.1038/nrdp.2015.2 27189141

[2] Volkmann ER Andreasson K Smith V . Systemic sclerosis. Lancet. (2023) 401:304–18. doi: 10.1016/S0140-6736(22)01692-0 PMC9892343 36442487

[3] Ingegnoli F Ughi N Mihai C . Update on the epidemiology, risk factors, and disease outcomes of systemic sclerosis. Best Pract Res Clin Rheumatol. (2018) 32:223–40. doi: 10.1016/j.berh.2018.08.005 30527428

[4] Elhai M Meune C Avouac J Kahan A Allanore Y . Trends in mortality in patients with systemic sclerosis over 40 years: a systematic review and meta-analysis of cohort studies. Rheumatol (Oxford). (2012) 51:1017–26. doi: 10.1093/rheumatology/ker269 21900368

[5] Tyndall AJ Bannert B Vonk M Airo P Cozzi F Carreira PE . Causes and risk factors for death in systemic sclerosis: a study from the EULAR Scleroderma Trials and Research (EUSTAR) database. Ann Rheum Dis. (2010) 69:1809–15. doi: 10.1136/ard.2009.114264 20551155

[6] Aozasa N Hatano M Saigusa R Nakamura K Takahashi T Toyama T . Clinical significance of endothelial vasodilatory function evaluated by EndoPAT in patients with systemic sclerosis. J Dermatol. (2020) 47:609–14. doi: 10.1111/1346-8138.15334 32232898

[7] Au K Singh MK Bodukam V Bae S Maranian P Ogawa R . Atherosclerosis in systemic sclerosis: a systematic review and meta-analysis. Arthritis Rheum. (2011) 63:2078–90. doi: 10.1002/art.30380 PMC3128188 21480189

[8] Patnaik E Lyons M Tran K Pattanaik D . Endothelial dysfunction in systemic sclerosis. Int J Mol Sci. (2023) 24:14385. doi: 10.3390/ijms241814385 37762689 PMC10531630

[9] Sciarra I Vasile M Carboni A Stefanantoni K Iannace N Angelelli C . Subclinical atherosclerosis in systemic sclerosis: Different risk profiles among patients according to clinical manifestations. Int J Rheum Dis. (2021) 24:502–9. doi: 10.1111/1756-185X.14002 33073534

[10] Psarras A Soulaidopoulos S Garyfallos A Kitas G Dimitroulas T . A critical view on cardiovascular risk in systemic sclerosis. Rheumatol Int. (2017) 37:85–95. doi: 10.1007/s00296-016-3530-3 27405985

[11] Dimitroulas T Baniotopoulos P Pagkopoulou E Soulaidopoulos S Nightingale P Sandoo A . Subclinical atherosclerosis in systemic sclerosis and rheumatoid arthritis: a comparative matched-cohort study. Rheumatol Int. (2020) 40:1997–2004. doi: 10.1007/s00296-020-04677-3 32772133

[12] Reiss AB Silverman A Khalfan M Vernice NA Kasselman LJ Carsons SE . Accelerated atherosclerosis in rheumatoid arthritis: mechanisms and treatment. Curr Pharm Des. (2019) 25:969–86. doi: 10.2174/1381612825666190430113212 31208307

[13] Kurmann RD Sandhu AS Crowson CS Matteson EL Osborn TG Warrington KJ . Cardiovascular risk factors and atherosclerotic cardiovascular events among incident cases of systemic sclerosis: results from a population-based cohort (1980-2016). Mayo Clin Proc. (2020) 95:1369–78. doi: 10.1016/j.mayocp.2019.12.015 PMC9719716 32622445

[14] Butt SA Jeppesen JL Torp-Pedersen C Sam F Gislason GH Jacobsen S . Cardiovascular manifestations of systemic sclerosis: A danish nationwide cohort study. J Am Heart Assoc. (2019) 8:e013405. doi: 10.1161/JAHA.119.013405 31446827 PMC6755829

[15] Napoli C de Nigris F Williams-Ignarro S Pignalosa O Sica V Ignarro LJ . Nitric oxide and atherosclerosis: an update. Nitric Oxide. (2006) 15:265–79. doi: 10.1016/j.niox.2006.03.011 16684613

[16] Falk E . Pathogenesis of atherosclerosis. J Am Coll Cardiol. (2006) 47:C7–12. doi: 10.1016/j.jacc.2005.09.068 16631513

[17] Jebari-Benslaiman S Galicia-Garcia U Larrea-Sebal A Olaetxea JR Alloza I Vandenbroeck K . Pathophysiology of atherosclerosis. Int J Mol Sci. (2022) 23:3346. doi: 10.3390/ijms23063346 35328769 PMC8954705

[18] Price DT Loscalzo J . Cellular adhesion molecules and atherogenesis. Am J Med. (1999) 107:85–97. doi: 10.1016/s0002-9343(99)00153-9 10403357

[19] Blankenberg S Barbaux S Tiret L . Adhesion molecules and atherosclerosis. Atherosclerosis. (2003) 170:191–203. doi: 10.1016/s0021-9150(03)00097-2 14612198

[20] Zhong L Simard MJ Huot J . Endothelial microRNAs regulating the NF-kappaB pathway and cell adhesion molecules during inflammation. FASEB J. (2018) 32:4070–84. doi: 10.1096/fj.201701536R 29565737

[21] Woodfin A Voisin MB Nourshargh S . PECAM-1: a multi-functional molecule in inflammation and vascular biology. Arterioscler Thromb Vasc Biol. (2007) 27:2514–23. doi: 10.1161/ATVBAHA.107.151456 17872453

[22] Bowden RA Ding ZM Donnachie EM Petersen TK Michael LH Ballantyne CM . Role of alpha4 integrin and VCAM-1 in CD18-independent neutrophil migration across mouse cardiac endothelium. Circ Res. (2002) 90:562–9. doi: 10.1161/01.res.0000013835.53611.97 11909820

[23] Kong DH Kim YK Kim MR Jang JH Lee S . Emerging roles of vascular cell adhesion molecule-1 (VCAM-1) in immunological disorders and cancer. Int J Mol Sci. (2018) 19:1057. doi: 10.3390/ijms19041057 29614819 PMC5979609

[24] Lawson C Wolf S . ICAM-1 signaling in endothelial cells. Pharmacol Rep. (2009) 61:22–32. doi: 10.1016/s1734-1140(09)70004-0 19307690

[25] Bui TM Wiesolek HL Sumagin R . ICAM-1: A master regulator of cellular responses in inflammation, injury resolution, and tumorigenesis. J Leukoc Biol. (2020) 108:787–99. doi: 10.1002/JLB.2MR0220-549R PMC7977775 32182390

[26] Dangerfield J Larbi KY Huang MT Dewar A Nourshargh S . PECAM-1 (CD31) homophilic interaction up-regulates alpha6beta1 on transmigrated neutrophils in *vivo* and plays a functional role in the ability of alpha6 integrins to mediate leukocyte migration through the perivascular basement membrane. J Exp Med. (2002) 196:1201–11. doi: 10.1084/jem.20020324 PMC2194111 12417630

[27] Sowparani S Mahalakshmi P Sweety JP Francis AP Dhanalekshmi UM Selvasudha N . Ubiquitous neural cell adhesion molecule (NCAM): potential mechanism and valorisation in cancer pathophysiology, drug targeting and molecular transductions. Mol Neurobiol. (2022) 59:5902–24. doi: 10.1007/s12035-022-02954-9 35831555

[28] Zhu K Xu Y Liu J Xu Q Ye H . Down syndrome cell adhesion molecule and its functions in neural development. Neurosci Bull. (2011) 27:45–52. doi: 10.1007/s12264-011-1045-1 21270903 PMC5560281

[29] Inoue M Ishida T Yasuda T Toh R Hara T Cangara HM . Endothelial cell-selective adhesion molecule modulates atherosclerosis through plaque angiogenesis and monocyte-endothelial interaction. Microvasc Res. (2010) 80:179–87. doi: 10.1016/j.mvr.2010.04.005 20406651

[30] Videm V Albrigtsen M . Soluble ICAM-1 and VCAM-1 as markers of endothelial activation. Scand J Immunol. (2008) 67:523–31. doi: 10.1111/j.1365-3083.2008.02029.x 18363595

[31] Villar J Muros M Cabrera-Benitez NE Valladares F Lopez-Hernandez M Flores C . Soluble platelet-endothelial cell adhesion molecule-1, a biomarker of ventilator-induced lung injury. Crit Care. (2014) 18:R41. doi: 10.1186/cc13754 24588994 PMC4057495

[32] Luc G Arveiler D Evans A Amouyel P Ferrieres J Bard JM . Circulating soluble adhesion molecules ICAM-1 and VCAM-1 and incident coronary heart disease: the PRIME Study. Atherosclerosis. (2003) 170:169–76. doi: 10.1016/s0021-9150(03)00280-6 12957696

[33] Kaur R Singh V Kumari P Singh R Chopra H Emran TB . Novel insights on the role of VCAM-1 and ICAM-1: Potential biomarkers for cardiovascular diseases. Ann Med Surg (Lond). (2022) 84:104802. doi: 10.1016/j.amsu.2022.104802 36408439 PMC9672401

[34] Troncoso MF Ortiz-Quintero J Garrido-Moreno V Sanhueza-Olivares F Guerrero-Moncayo A Chiong M . VCAM-1 as a predictor biomarker in cardiovascular disease. Biochim Biophys Acta Mol Basis Dis. (2021) 1867:166170. doi: 10.1016/j.bbadis.2021.166170 34000374

[35] Singh V Kaur R Kumari P Pasricha C Singh R . ICAM-1 and VCAM-1: Gatekeepers in various inflammatory and cardiovascular disorders. Clin Chim Acta. (2023) 548:117487. doi: 10.1016/j.cca.2023.117487 37442359

[36] Sahebkar A Morris DR Biros E Golledge J . Association of single nucleotide polymorphisms in the gene encoding platelet endothelial cell adhesion molecule-1 with the risk of myocardial infarction: a systematic review and meta-analysis. Thromb Res. (2013) 132:227–33. doi: 10.1016/j.thromres.2013.07.007 23906939

[37] Ren HY Khera A de Lemos JA Ayers CR Rohatgi A . Soluble endothelial cell-selective adhesion molecule and incident cardiovascular events in a multiethnic population. Am Heart J. (2017) 191:55–61. doi: 10.1016/j.ahj.2017.06.008 28888270 PMC5657571

[38] Rohatgi A Owens AW Khera A Ayers CR Banks K Das SR . Differential associations between soluble cellular adhesion molecules and atherosclerosis in the Dallas Heart Study: a distinct role for soluble endothelial cell-selective adhesion molecule. Arterioscler Thromb Vasc Biol. (2009) 29:1684–90. doi: 10.1161/ATVBAHA.109.190553 PMC2771407 19759376

[39] Galkina E Ley K . Vascular adhesion molecules in atherosclerosis. Arterioscler Thromb Vasc Biol. (2007) 27:2292–301. doi: 10.1161/ATVBAHA.107.149179 17673705

[40] Maitre JL Heisenberg CP . Three functions of cadherins in cell adhesion. Curr Biol. (2013) 23:R626–33. doi: 10.1016/j.cub.2013.06.019 PMC3722483 23885883

[41] Ley K . The role of selectins in inflammation and disease. Trends Mol Med. (2003) 9:263–8. doi: 10.1016/s1471-4914(03)00071-6 12829015

[42] McEver RP . Selectins: initiators of leucocyte adhesion and signalling at the vascular wall. Cardiovasc Res. (2015) 107:331–9. doi: 10.1093/cvr/cvv154 PMC4592324 25994174

[43] Tvaroska I Selvaraj C Koca J . Selectins-the two dr. Jekyll and mr. Hyde faces of adhesion molecules-A review. Molecules. (2020) 25:2835. doi: 10.3390/molecules25122835 32575485 PMC7355470

[44] McEver RP Zhu C . Rolling cell adhesion. Annu Rev Cell Dev Biol. (2010) 26:363–96. doi: 10.1146/annurev.cellbio.042308.113238 PMC3557855 19575676

[45] Ivetic A Hoskins Green HL Hart SJ . L-selectin: A major regulator of leukocyte adhesion, migration and signaling. Front Immunol. (2019) 10:1068. doi: 10.3389/fimmu.2019.01068 31139190 PMC6527602

[46] Ridker PM Buring JE Rifai N . Soluble P-selectin and the risk of future cardiovascular events. Circulation. (2001) 103:491–5. doi: 10.1161/01.cir.103.4.491 11157711

[47] Roldan V Marin F Lip GY Blann AD . Soluble E-selectin in cardiovascular disease and its risk factors. A review of the literature. Thromb Haemost. (2003) 90:1007–20. doi: 10.1160/TH02-09-0083 14652631

[48] Bielinski SJ Berardi C Decker PA Kirsch PS Larson NB Pankow JS . P-selectin and subclinical and clinical atherosclerosis: the Multi-Ethnic Study of Atherosclerosis (MESA). Atherosclerosis. (2015) 240:3–9. doi: 10.1016/j.atherosclerosis.2015.02.036 25744700 PMC4397161

[49] Eikendal ALM Bots ML Gohar A Lutgens E Hoefer IE den Ruijter HM . Circulating levels of P-selectin and E-selectin relate to cardiovascular magnetic resonance-derived aortic characteristics in young adults from the general population, a cross-sectional study. J Cardiovasc Magn Reson. (2018) 20:54. doi: 10.1186/s12968-018-0473-8 30068374 PMC6090925

[50] de Almeida-Pititto B Ribeiro-Filho FF Bittencourt MS Lotufo PA Bensenor I Ferreira SR . Usefulness of circulating E-selectin to early detection of the atherosclerotic process in the Brazilian Longitudinal Study of Adult Health (ELSA-Brasil). Diabetol Metab Syndr. (2016) 8:19. doi: 10.1186/s13098-016-0133-9 26949419 PMC4778299

[51] Finney AC Stokes KY Pattillo CB Orr AW . Integrin signaling in atherosclerosis. Cell Mol Life Sci. (2017) 74:2263–82. doi: 10.1007/s00018-017-2490-4 PMC5427000 28246700

[52] Ferrell PD Oristian KM Puranam I Pizzo SV . Serum pro-N-cadherin is a marker of subclinical heart failure in the general population. J Am Heart Assoc. (2023) 12:e028234. doi: 10.1161/JAHA.122.028234 36892069 PMC10111553

[53] Moola S Munn Z Tufanaru C Aromataris E Sears K Sfetcu R . Systematic reviews of etiology and risk. In: Aromataris E Munn Z , editors. Joanna Briggs Institute Reviewer’s Manual. Johanna Briggs Institute, Adelaide, Australia (2017).

[54] Balshem H Helfand M Schunemann HJ Oxman AD Kunz R Brozek J . GRADE guidelines: 3. Rating the quality of evidence. J Clin Epidemiol. (2011) 64:401–6. doi: 10.1016/j.jclinepi.2010.07.015 21208779

[55] Page MJ McKenzie JE Bossuyt PM Boutron I Hoffmann TC Mulrow CD . The PRISMA 2020 statement: an updated guideline for reporting systematic reviews. BMJ. (2021) 372:n71. doi: 10.1136/bmj.n71 33782057 PMC8005924

[56] Wan X Wang W Liu J Tong T . Estimating the sample mean and standard deviation from the sample size, median, range and/or interquartile range. BMC Med Res Methodol. (2014) 14:135. doi: 10.1186/1471-2288-14-135 25524443 PMC4383202

[57] Higgins JP Thompson SG . Quantifying heterogeneity in a meta-analysis. Stat Med. (2002) 21:1539–58. doi: 10.1002/sim.1186 12111919

[58] Higgins JP Thompson SG Deeks JJ Altman DG . Measuring inconsistency in meta-analyses. BMJ. (2003) 327:557–60. doi: 10.1136/bmj.327.7414.557 PMC192859 12958120

[59] Tobias A . Assessing the influence of a single study in the meta-analysis estimate. Stata Tech Bull. (1999) 47:15–7.

[60] Begg CB Mazumdar M . Operating characteristics of a rank correlation test for publication bias. Biometrics. (1994) 50:1088–101. doi: 10.2307/2533446 7786990

[61] Sterne JA Egger M . Funnel plots for detecting bias in meta-analysis: guidelines on choice of axis. J Clin Epidemiol. (2001) 54:1046–55. doi: 10.1016/s0895-4356(01)00377-8 11576817

[62] Duval S Tweedie R . Trim and fill: A simple funnel-plot-based method of testing and adjusting for publication bias in meta-analysis. Biometrics. (2000) 56:455–63. doi: 10.1111/j.0006-341x.2000.00455.x 10877304

[63] Carson CW Beall LD Hunder GG Johnson CM Newman W . Serum ELAM-1 is increased in vasculitis, scleroderma, and systemic lupus erythematosus. J Rheumatol. (1993) 20:809–14. 7687701

[64] Sfikakis PP Tesar J Baraf H Lipnick R Klipple G Tsokos GC . Circulating intercellular adhesion molecule-1 in patients with systemic sclerosis. Clin Immunol Immunopathol. (1993) 68:88–92. doi: 10.1006/clin.1993.1100 8099861

[65] Kiener H Graninger W Machold K Aringer M Graninger WB . Increased levels of circulating intercellular adhesion molecule-1 in patients with systemic sclerosis. Clin Exp Rheumatol. (1994) 12:483–7. 7842528

[66] Blann AD Herrick A Jayson MI . Altered levels of soluble adhesion molecules in rheumatoid arthritis, vasculitis and systemic sclerosis. Br J Rheumatol. (1995) 34:814–9. doi: 10.1093/rheumatology/34.9.814 7582719

[67] Gruschwitz MS Hornstein OP von Den Driesch P . Correlation of soluble adhesion molecules in the peripheral blood of scleroderma patients with their in *situ* expression and with disease activity. Arthritis Rheum. (1995) 38:184–9. doi: 10.1002/art.1780380206 7848308

[68] Blann AD Sanders PA Herrick A Jayson MI . Soluble L-selectin in the connective tissue diseases. Br J Haematol. (1996) 95:192–4. doi: 10.1046/j.1365-2141.1996.7562378.x 8857959

[69] Ihn H Sato S Fujimoto M Kikuchi K Kadono T Tamaki K . Circulating intercellular adhesion molecule-1 in the sera of patients with systemic sclerosis: enhancement by inflammatory cytokines. Br J Rheumatol. (1997) 36:1270–5. doi: 10.1093/rheumatology/36.12.1270 9448587

[70] Ihn H Sato S Fujimoto M Takehara K Tamaki K . Increased serum levels of soluble vascular cell adhesion molecule-1 and E-selectin in patients with systemic sclerosis. Br J Rheumatol. (1998) 37:1188–92. doi: 10.1093/rheumatology/37.11.1188 9851267

[71] Majewski S Wojas-Pelc A Malejczyk M Szymanska E Jablonska S . Serum levels of soluble TNF alpha receptor type I and the severity of systemic sclerosis. Acta Derm Venereol. (1999) 79:207–10. doi: 10.1080/000155599750010986 10384918

[72] Sfikakis PP Charalambopoulos D Vaiopoulos G Mavrikakis M . Circulating P- and L-selectin and T-lymphocyte activation and patients with autoimmune rheumatic diseases. Clin Rheumatol. (1999) 18:28–32. doi: 10.1007/s100670050047 10088945

[73] Andersen GN Mincheva-Nilsson L Kazzam E Nyberg G Klintland N Petersson AS . Assessment of vascular function in systemic sclerosis: Indications of the development of nitrate tolerance as a result of enhanced endothelial nitric oxide production. Arthritis Rheumatism. (2002) 46:1324–32. doi: 10.1002/art.10191 12115240

[74] Macko RF Gelber AC Young BA Lowitt MH White B Wigley FM . Increased circulating concentrations of the counteradhesive proteins SPARC and thrombospondin-1 in systemic sclerosis (scleroderma). Relationship to platelet and endothelial cell activation. J Rheumatol. (2002) 29:2565–70. 12465153

[75] Blann AD Constans J Carpentier P Renard M Satger B Guérin V . Soluble P selectin in systemic sclerosis: relationship with von Willebrand factor, autoantibodies and diffuse or localised/limited disease. Thromb Res. (2003) 109:203–6. doi: 10.1016/s0049-3848(03)00209-3 12757775

[76] Cerinic MM Valentini G Sorano GG D’Angelo S Cuomo G Fenu L . Blood coagulation, fibrinolysis, and markers of endothelial dysfunction in systemic sclerosis. Semin Arthritis Rheumatism. (2003) 32:285–95. doi: 10.1053/sarh.2002.50011 12701039

[77] Zamzam ML Yassin MM Sallam MM . Implication of intercellular adhesion molecule-1 (ICAM-1) and serum N(G)-hydroxy-L-arginine (L-NHA) in the pathogenesis of systemic sclerosis. Egypt J Immunol. (2003) 10:27–38. 15719609

[78] Allanore Y Borderie D Lemarechal H Ekindjian OG Kahan A . Nifedipine decreases sVCAM-1 concentrations and oxidative stress in systemic sclerosis but does not affect the concentrations of vascular endothelial growth factor or its soluble receptor 1. Arthritis Res Ther. (2004) 6:R309–14. doi: 10.1186/ar1183 PMC464873 15225366

[79] Ateş A Kinikli G Turgay M Duman M . Serum-soluble selectin levels in patients with rheumatoid arthritis and systemic sclerosis. Scandinavian J Immunol. (2004) 59:315–20. doi: 10.1111/j.0300-9475.2004.01389.x 15030584

[80] Kuryliszyn-Moskal A Klimiuk PA Sierakowski S . Soluble adhesion molecules (sVCAM-1, sE-selectin), vascular endothelial growth factor (VEGF) and endothelin-1 in patients with systemic sclerosis: relationship to organ systemic involvement. Clin Rheumatol. (2004) 24:111–6. doi: 10.1007/s10067-004-0987-3 15349798

[81] Dovio A Data V Carignola R Calzolari G Vitetta R Ventura M . Circulating osteoprotegerin and soluble RANK ligand in systemic sclerosis. J Rheumatol. (2008) 35:2206–13. doi: 10.3899/jrheum.080192 18843778

[82] Hettema ME Zhang D de Leeuw K Stienstra Y Smit AJ Kallenberg CGM . Early atherosclerosis in systemic sclerosis and its relation to disease or traditional risk factors. Arthritis Res Ther. (2008) 10:R49. doi: 10.1186/ar2408 18439295 PMC2453769

[83] Iannone F Riccardi MT Guiducci S Bizzoca R Cinelli M Matucci-Cerinic M . Bosentan regulates the expression of adhesion molecules on circulating T cells and serum soluble adhesion molecules in systemic sclerosis-associated pulmonary arterial hypertension. Ann Rheumatic Dis. (2008) 67:1121–6. doi: 10.1136/ard.2007.080424 PMC2564790 18029384

[84] Nomura S Inami N Ozaki Y Kagawa H Fukuhara S . Significance of microparticles in progressive systemic sclerosis with interstitial pneumonia. Platelets. (2009) 19:192–8. doi: 10.1080/09537100701882038 18432520

[85] Minier T Nagy Z Balint Z Farkas H Radics J Kumanovics G . Construct validity evaluation of the European Scleroderma Study Group activity index, and investigation of possible new disease activity markers in systemic sclerosis. Rheumatology. (2010) 49:1133–45. doi: 10.1093/rheumatology/keq022 20236952

[86] Olewicz-Gawlik A Danczak-Pazdrowska A Klama K Silny W Prokop J Mackiewicz S . Blood serum levels of amino-terminal pro-C-type natriuretic peptide in patients with systemic sclerosis. Connective Tissue Res. (2010) 51:83–7. doi: 10.3109/03008200903056168 20001845

[87] Alzawawy AI Suliman I Hamimi A Elsawy N Albordiny MM . Serum soluble vascular cell adhesion molecule-1 (sVCAM-1) in scleroderma patients and its relation to pulmonary involvement and disease activity. Egyptian Rheumatologist. (2011) 33:21–6. doi: 10.1016/j.ejr.2010.06.001

[88] Riccieri V Stefanantoni K Vasile M Macri V Sciarra I Iannace N . Abnormal plasma levels of different angiogenic molecules are associated with different clinical manifestations in patients with systemic sclerosis. Clin Exp Rheumatol. (2011) 29:S46–52. 21586218

[89] Dunne JV van Eeden SF Keen KJ . L-selectin and skin damage in systemic sclerosis. PloS One. (2012) 7:e44814. doi: 10.1371/journal.pone.0044814 23028631 PMC3441480

[90] Aydoğdu E Pamuk ÖN Dönmez S Pamuk GE . Decreased interleukin-20 level in patients with systemic sclerosis: are they related with angiogenesis? Clin Rheumatol. (2013) 32:1599–603. doi: 10.1007/s10067-013-2317-0 23812620

[91] Iversen LV Østergaard O Ullman S Nielsen CT Halberg P Karlsmark T . Circulating microparticles and plasma levels of soluble E- and P-selectins in patients with systemic sclerosis. Scandinavian J Rheumatol. (2013) 42:473–82. doi: 10.3109/03009742.2013.796403 24016306

[92] Cossu M Andracco R Santaniello A Marchini M Severino A Caronni M . Serum levels of vascular dysfunction markers reflect disease severity and stage in systemic sclerosis patients. Rheumatology. (2016) 55:1112–6. doi: 10.1093/rheumatology/kew017 26989111

[93] Yalçınkaya Y Adın- Çınar S Artim-Esen B Kamalı S Pehlivan Ö Öcal L . Capillaroscopic findings and vascular biomarkers in systemic sclerosis: Association of low CD40L levels with late scleroderma pattern. Microvascular Res. (2016) 108:17–21. doi: 10.1016/j.mvr.2016.07.002 27392528

[94] Delle Sedie A Riente L Maggiorini L Pratesi F Tavoni A Migliorini P . Potential biomarkers in patients with systemic sclerosis. Int J Rheumatic Dis. (2018) 21:261–5. doi: 10.1111/1756-185x.13196 29024388

[95] Thakkar V Patterson KA Stevens W Wilson M Roddy J Sahhar J . Increased serum levels of adhesion molecules ICAM-1 and VCAM-1 in systemic sclerosis are not specific for pulmonary manifestations. Clin Rheumatol. (2018) 37:1563–71. doi: 10.1007/s10067-018-4081-7 29687288

[96] Wodok-Wieczorek K Salwowska N Syguła E Wodok A Wcisło-Dziadecka D Bebenek K . The correlation between serum E-selectin levels and soluble interleukin-2 receptors with relation to disease activity in localized scleroderma. Adv Dermatol Allergology. (2018) 35:614–9. doi: 10.5114/ada.2018.77613 PMC6320493 30618531

[97] Hegazy GA Shaker O Sayed S Elzaher AA Fathy K Wahby I . Biomarkers of Systemic Lupus Erythematosus and Systemic Sclerosis diseases activity in a sample of Egyptian patients:Soluble Intercellular Adhesion Molecule-1 and Soluble Interleukin-2 Receptor, Case Control Study. Biomed Pharmacol J. (2019) 12:1207–16. doi: 10.13005/bpj/1750

[98] Pacholczak-Madej R Kuszmiersz P Bazan-Socha S Kosałka-Węgiel J Iwaniec T Zaręba L . Endothelial dysfunction in patients with systemic sclerosis. Adv Dermatol Allergology. (2020) 37:495–502. doi: 10.5114/ada.2019.83501 PMC7507157 32994769

[99] Al-Omary Obadeh M Bondar S . Endothelial dysfunction and pathogenetic phenotypes of localized scleroderma. Georgian Med News. (2021) 319:102–8. 34749332

[100] Kuszmiersz P Pacholczak-Madej R Siwiec A Celinska-Lowenhoff M Iwaniec T Kosalka-Wegiel J . Thrombin generation potential is enhanced in systemic sclerosis: impact of selected endothelial biomarkers. Clin Exp Rheumatol. (2021) 39 Suppl 131:13–9. doi: 10.55563/clinexprheumatol/d03dnc 33769265

[101] Stern EP Unwin R Burns A Ong VH Denton CP . Exploring molecular pathology of chronic kidney disease in systemic sclerosis by analysis of urinary and serum proteins. Rheumatol Adv Pract. (2021) 5:rkaa083. doi: 10.1093/rap/rkaa083 33604504 PMC7878848

[102] Brezovec N Perdan-Pirkmajer K Kuret T Burja B Sodin-Šemrl S Čučnik S . Increased L-selectin on monocytes is linked to the autoantibody profile in systemic sclerosis. Int J Mol Sci. (2022) 23:2233. doi: 10.3390/ijms23042233 35216350 PMC8880182

[103] Colic J Pruner I Damjanov N Pekmezovic T Sefik-Bukilica M Antovic A . Impaired fibrinolysis is linked with digital vasculopathy and onset of new digital ulcers in systemic sclerosis. J Rheumatol. (2022) 49:598–606. doi: 10.3899/jrheum.210931 35169064

[104] Jee AS Stewart I Youssef P Adelstein S Lai D Hua S . A composite serum biomarker index for the diagnosis of systemic sclerosis-associated interstitial lung disease: A multicenter, observational cohort study. Arthritis Rheumatol. (2023) 75:1424–33. doi: 10.1002/art.42491 36908055

[105] Corrado A Mansueto N Correale M Rella V Tricarico L Altomare A . Flow Mediated Dilation in Systemic Sclerosis: Association with clinical findings, capillaroscopic patterns and endothelial circulating markers. Vasc Pharmacol. (2024) 154:107252. doi: 10.1016/j.vph.2023.107252 38061409

[106] Cohen J . Statistical power analysis. Curr Dir Psychol Sci. (1992) 1:98–101. doi: 10.1111/1467-8721.ep10768783

[107] Jayson MI . The micro-circulation in systemic sclerosis. Clin Exp Rheumatol. (1984) 2:85–91. 6398169

[108] Lemmers JMJ Velauthapillai A van Herwaarden N Vonk MC . Change of the microvascularization in systemic sclerosis, a matter of air. Best Pract Res Clin Rheumatol. (2021) 35:101683. doi: 10.1016/j.berh.2021.101683 33814313

[109] Hsieh MC Chen HH Chou TY Su TW Lin CL Kao CH . Association between systemic sclerosis and peripheral arterial disease: a nationwide observation retrospective claim records cohort study in Taiwan. BMJ Open. (2021) 11:e048149. doi: 10.1136/bmjopen-2020-048149 PMC8483053 34588244

[110] Cannarile F Valentini V Mirabelli G Alunno A Terenzi R Luccioli F . Cardiovascular disease in systemic sclerosis. Ann Transl Med. (2015) 3:8. doi: 10.3978/j.issn.2305-5839.2014.12.12 25705640 PMC4293487

[111] Cockerill G Xu Q . Atherosclerosis. In: Fitridge R Thompson M , editors. Mechanisms of Vascular Disease: A Reference Book for Vascular Specialists. Adelaide (AU): University of Adelaide Press. (2011). 30484990

[112] Neidhart M Kuchen S Distler O Bruhlmann P Michel BA Gay RE . Increased serum levels of antibodies against human cytomegalovirus and prevalence of autoantibodies in systemic sclerosis. Arthritis Rheum. (1999) 42:389–92. doi: 10.1002/1529-0131(199902)42:2<389::AID-ANR23>3.0.CO;2-P 10025936

[113] Farina A Rosato E York M Gewurz BE Trojanowska M Farina GA . Innate immune modulation induced by EBV lytic infection promotes endothelial cell inflammation and vascular injury in scleroderma. Front Immunol. (2021) 12:651013. doi: 10.3389/fimmu.2021.651013 33953718 PMC8089375

[114] Maehara T Kaneko N Perugino CA Mattoo H Kers J Allard-Chamard H . Cytotoxic CD4+ T lymphocytes may induce endothelial cell apoptosis in systemic sclerosis. J Clin Invest. (2020) 130:2451–64. doi: 10.1172/JCI131700 PMC7190971 31990684

[115] Ihn H Sato S Fujimoto M Igarashi A Yazawa N Kubo M . Characterization of autoantibodies to endothelial cells in systemic sclerosis (SSc): association with pulmonary fibrosis. Clin Exp Immunol. (2000) 119:203–9. doi: 10.1046/j.1365-2249.2000.01115.x PMC1905540 10606984

[116] Kahaleh B . The microvascular endothelium in scleroderma. Rheumatol (Oxford). (2008) 47 Suppl 5:v14–5. doi: 10.1093/rheumatology/ken279 18784128

[117] Ishii N Matsumura T Shimoda S Araki E . Anti-atherosclerotic potential of dihydropyridine calcium channel blockers. J Atheroscler Thromb. (2012) 19:693–704. doi: 10.5551/jat.12450 22653165

[118] Zinellu A Mangoni AA . Systematic review and meta-analysis of the effect of statins on circulating E-selectin, L-selectin, and P-selectin. Biomedicines. (2021) 9:1707. doi: 10.3390/biomedicines9111707 34829936 PMC8615864

[119] Zinellu A Mangoni AA . A systematic review and meta-analysis of the effect of statin treatment on sVCAM-1 and sICAM-1. Expert Rev Clin Pharmacol. (2022) 15:601–20. doi: 10.1080/17512433.2022.2072294 35485866

[120] Zouki C Baron C Fournier A Filep JG . Endothelin-1 enhances neutrophil adhesion to human coronary artery endothelial cells: role of ET(A) receptors and platelet-activating factor. Br J Pharmacol. (1999) 127:969–79. doi: 10.1038/sj.bjp.0702593 PMC1566081 10433505

[121] Ibrahim MA Haleem M AbdelWahab SA Abdel-Aziz AM . Sildenafil ameliorates Alzheimer disease via the modulation of vascular endothelial growth factor and vascular cell adhesion molecule-1 in rats. Hum Exp Toxicol. (2021) 40:596–607. doi: 10.1177/0960327120960775 32959702

[122] Ahluwalia A Foster P Scotland RS McLean PG Mathur A Perretti M . Antiinflammatory activity of soluble guanylate cyclase: cGMP-dependent down-regulation of P-selectin expression and leukocyte recruitment. Proc Natl Acad Sci U.S.A. (2004) 101:1386–91. doi: 10.1073/pnas.0304264101 PMC337062 14742866

[123] Boehme MW Gao IK Norden C Lemmel EM . Decrease in circulating endothelial cell adhesion molecule and thrombomodulin levels during oral iloprost treatment in rheumatoid arthritis patients: preliminary results. Rheumatol Int. (2006) 26:340–7. doi: 10.1007/s00296-004-0563-9 15700117

