# Supplementary figures and images for: Circulating cell adhesion molecules in systemic sclerosis: a systematic review and meta-analysis
Source: Front Immunol. 2024 Aug 21;15:1438302. doi: 10.3389/fimmu.2024.1438302 (PMC11371573; doi:10.3389/fimmu.2024.1438302)

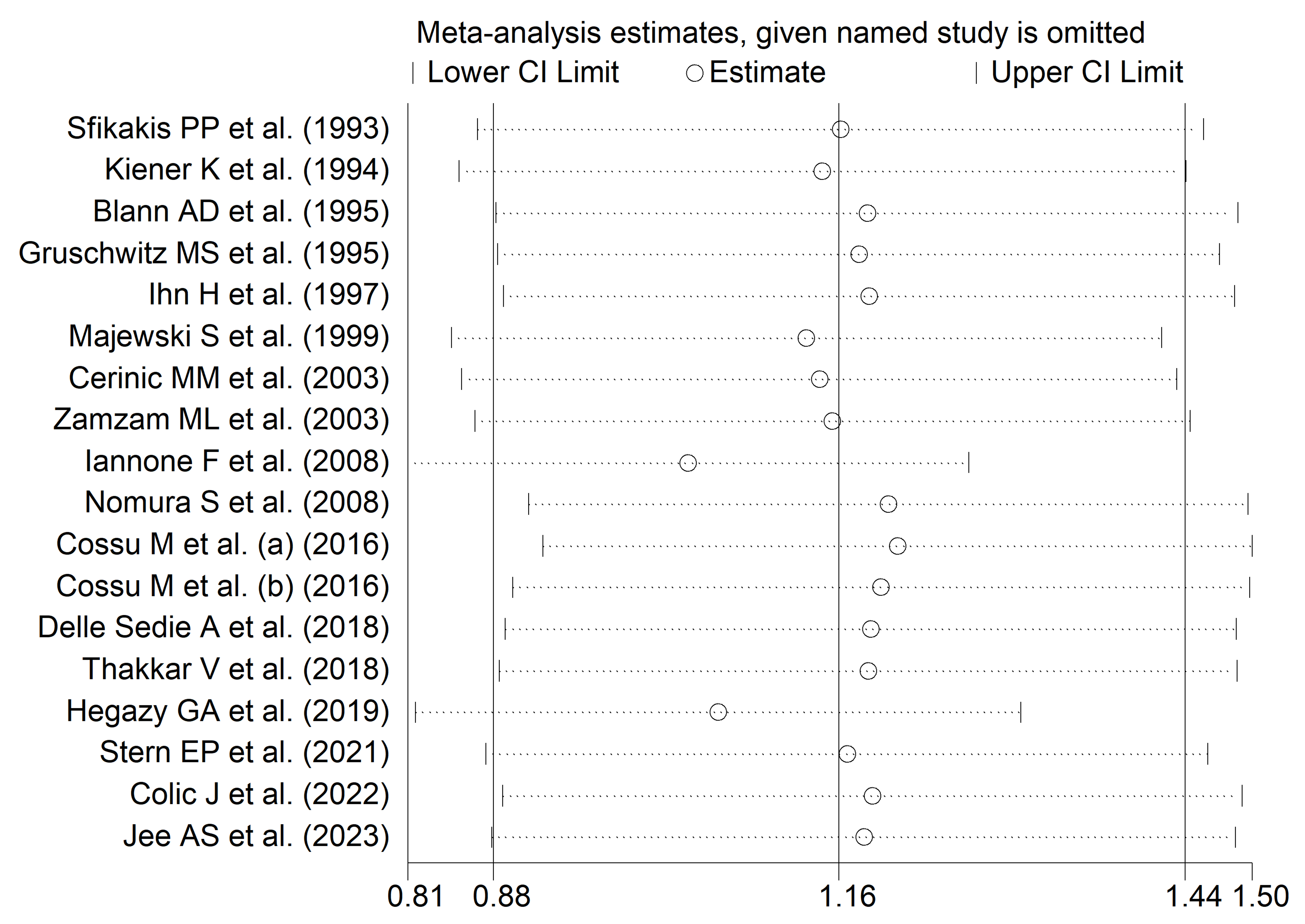

Supplement: Supplementary Figure 1 — Sensitivity analysis of the association between ICAM-1 concentrations and SSc. [file Image1.tif]

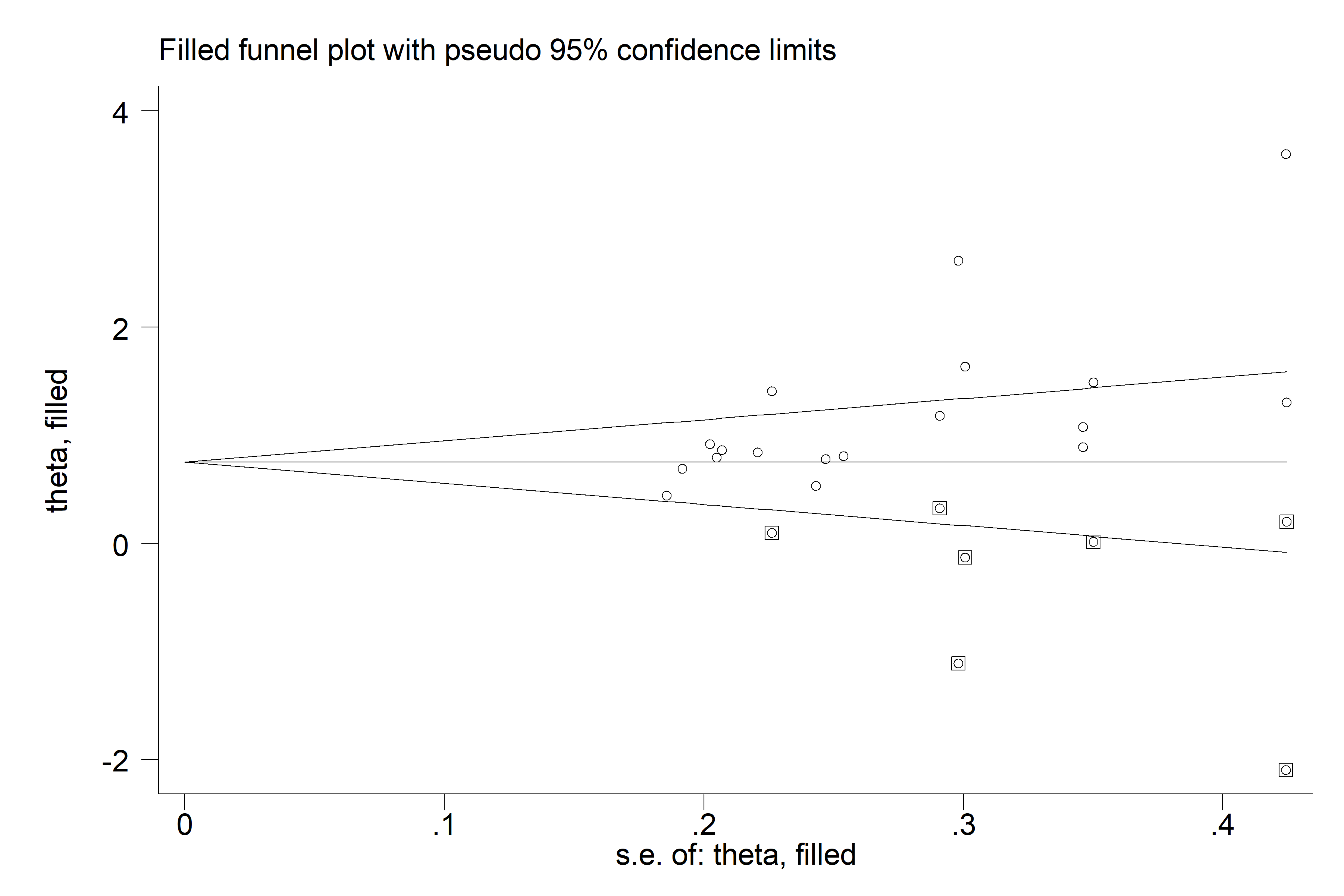

Supplement: Supplementary Figure 2 — Funnel plot of studies investigating the association between ICAM-1 concentrations and SSc after “trimming-and-filling”. Dummy studies and genuine studies are represented by enclosed circles and free circles, respectively. [file Image2.tif]

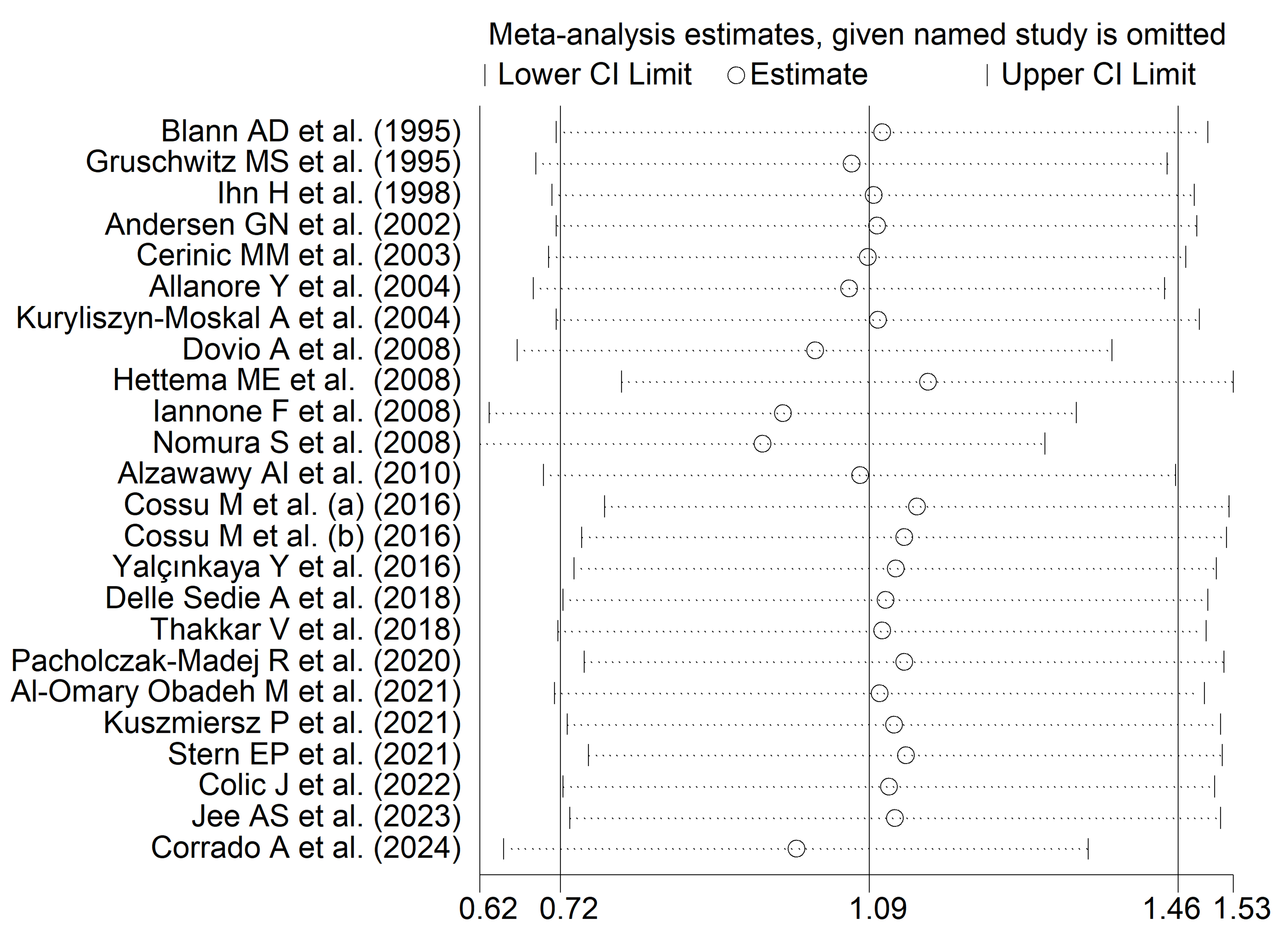

Supplement: Supplementary Figure 3 — Sensitivity analysis of the association between VCAM-1 concentrations and SSc. [file Image3.tif]

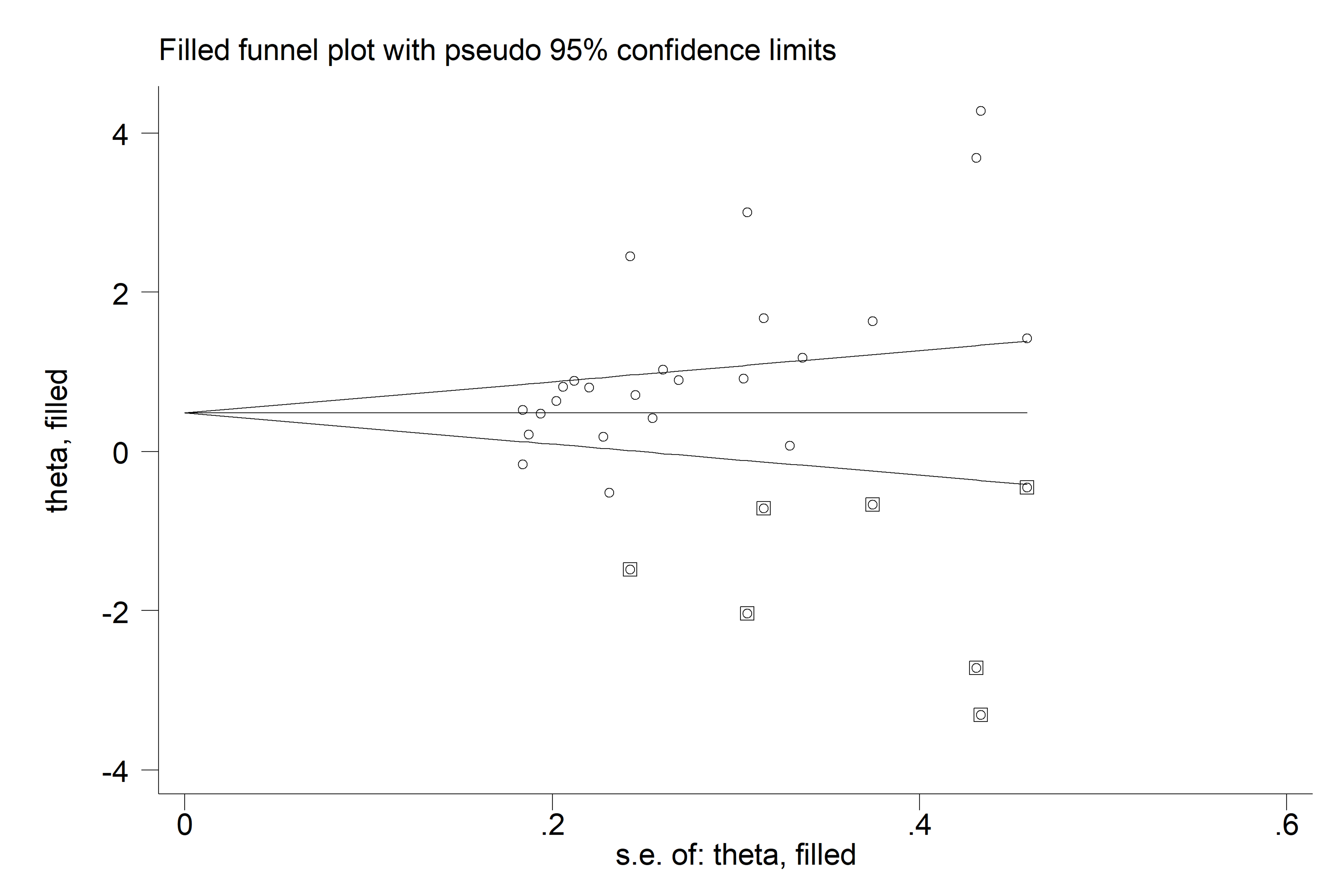

Supplement: Supplementary Figure 4 — Funnel plot of studies investigating the association between VCAM-1 concentrations and SSc after “trimming-and-filling”. Dummy studies and genuine studies are represented by enclosed circles and free circles, respectively. [file Image4.tif]

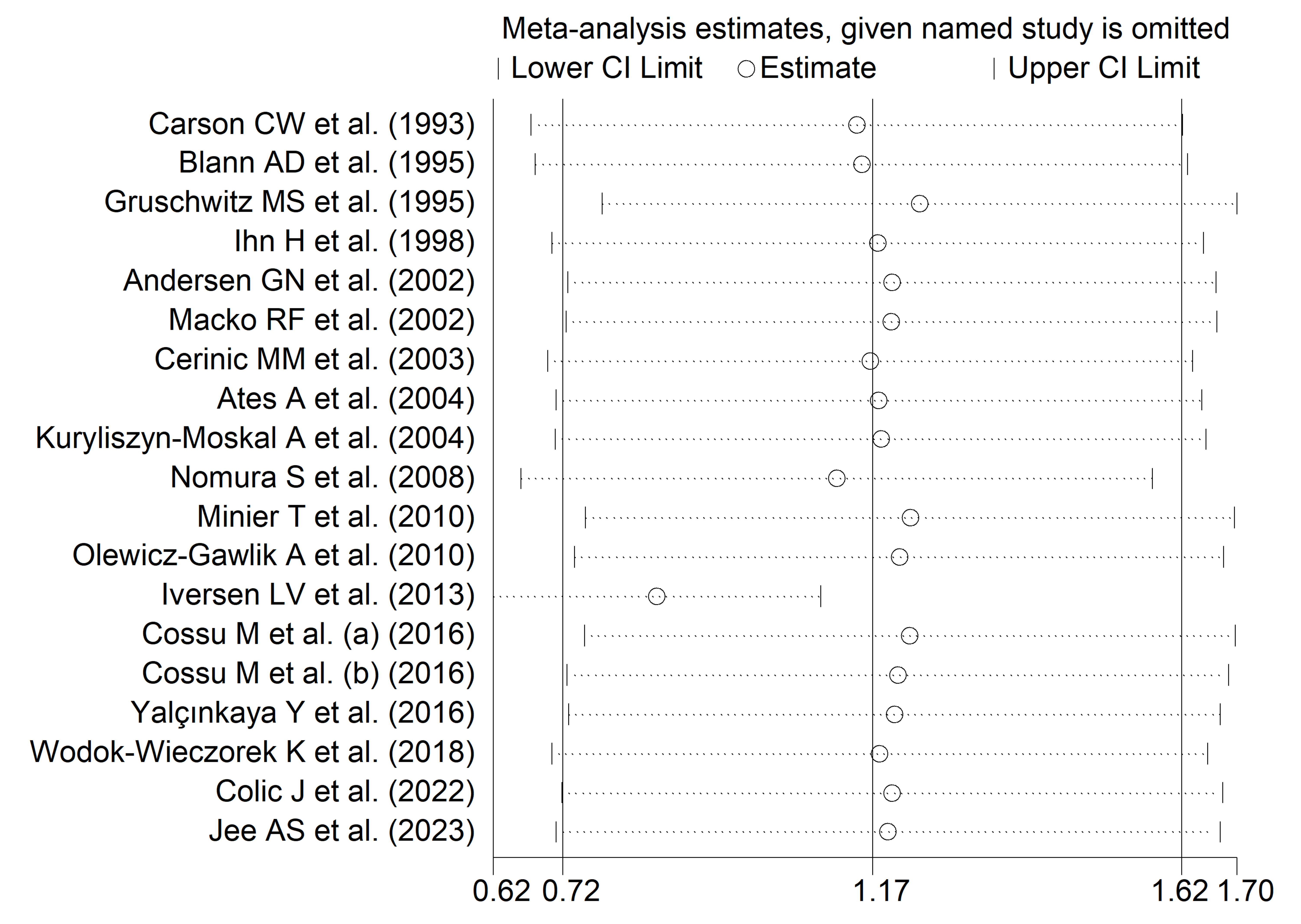

Supplement: Supplementary Figure 5 — Sensitivity analysis of the association between E-selectin concentrations and SSc. [file Image5.tif]

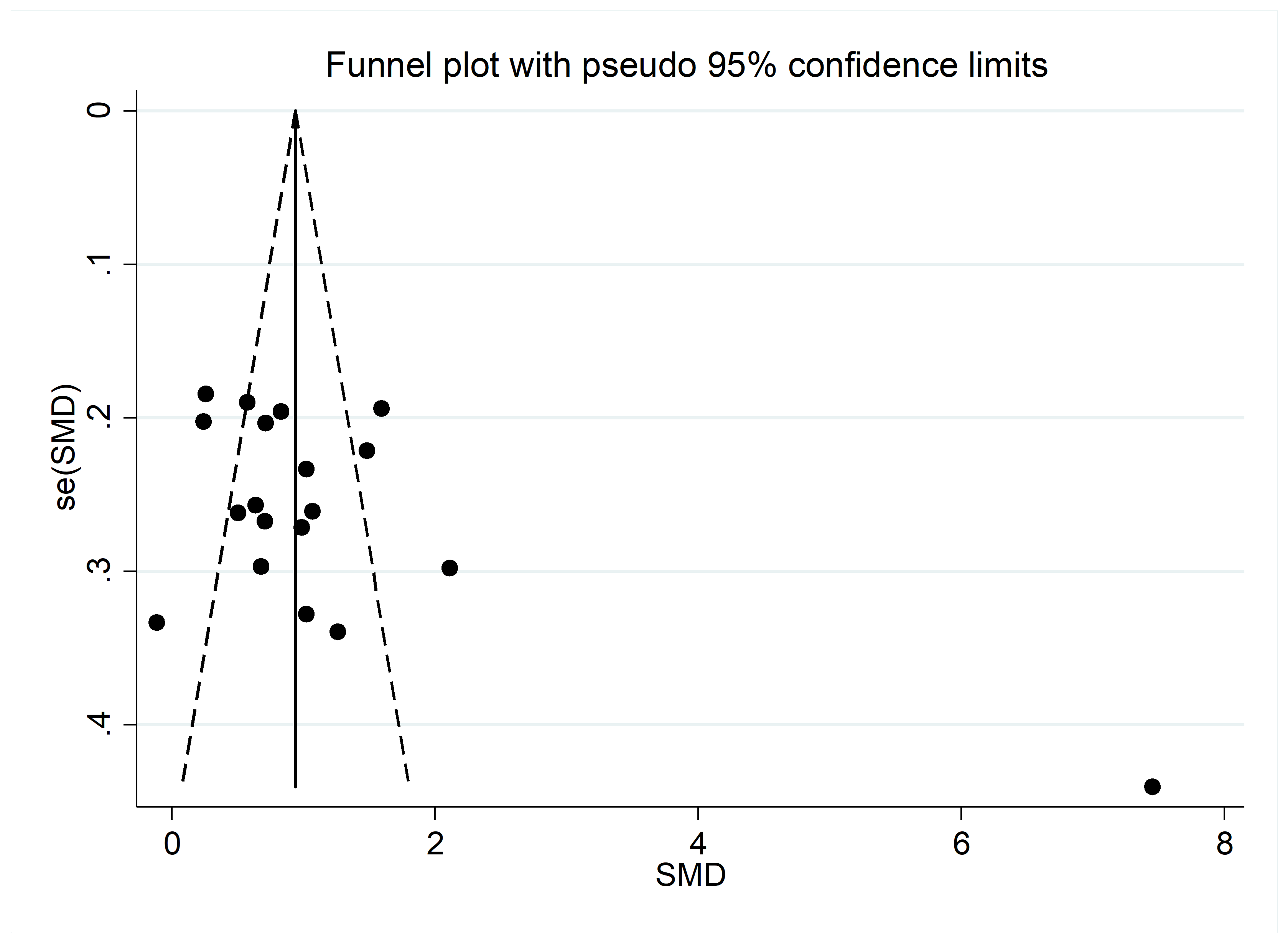

Supplement: Supplementary Figure 6 — Funnel plot of studies investigating the association between E-selectin concentrations and SSc. [file Image6.tif]

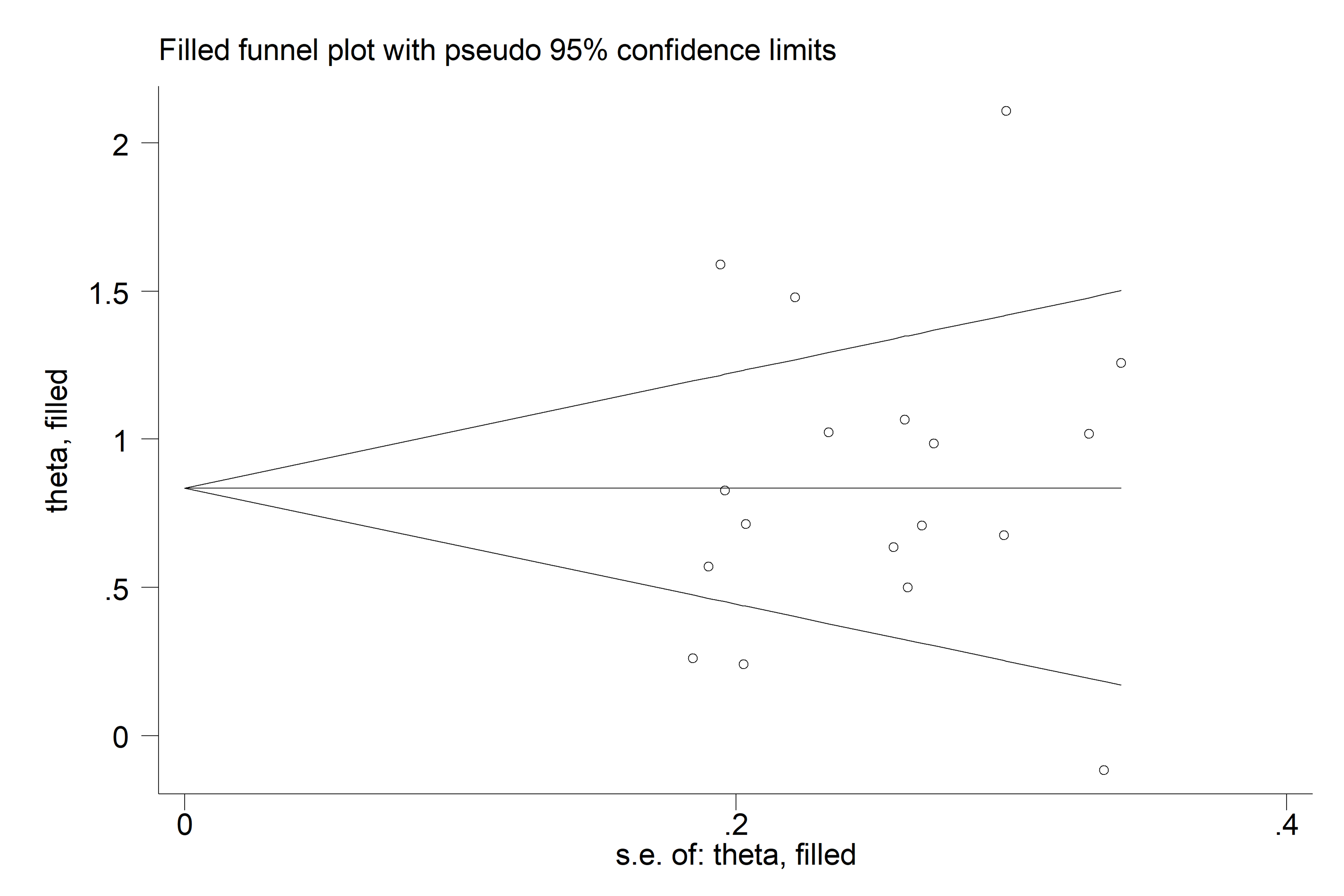

Supplement: Supplementary Figure 7 — Funnel plot of studies investigating the association between E-selectin concentrations and SSc after “trimming-and-filling”. Dummy studies and genuine studies are represented by enclosed circles and free circles, respectively. [file Image7.tif]

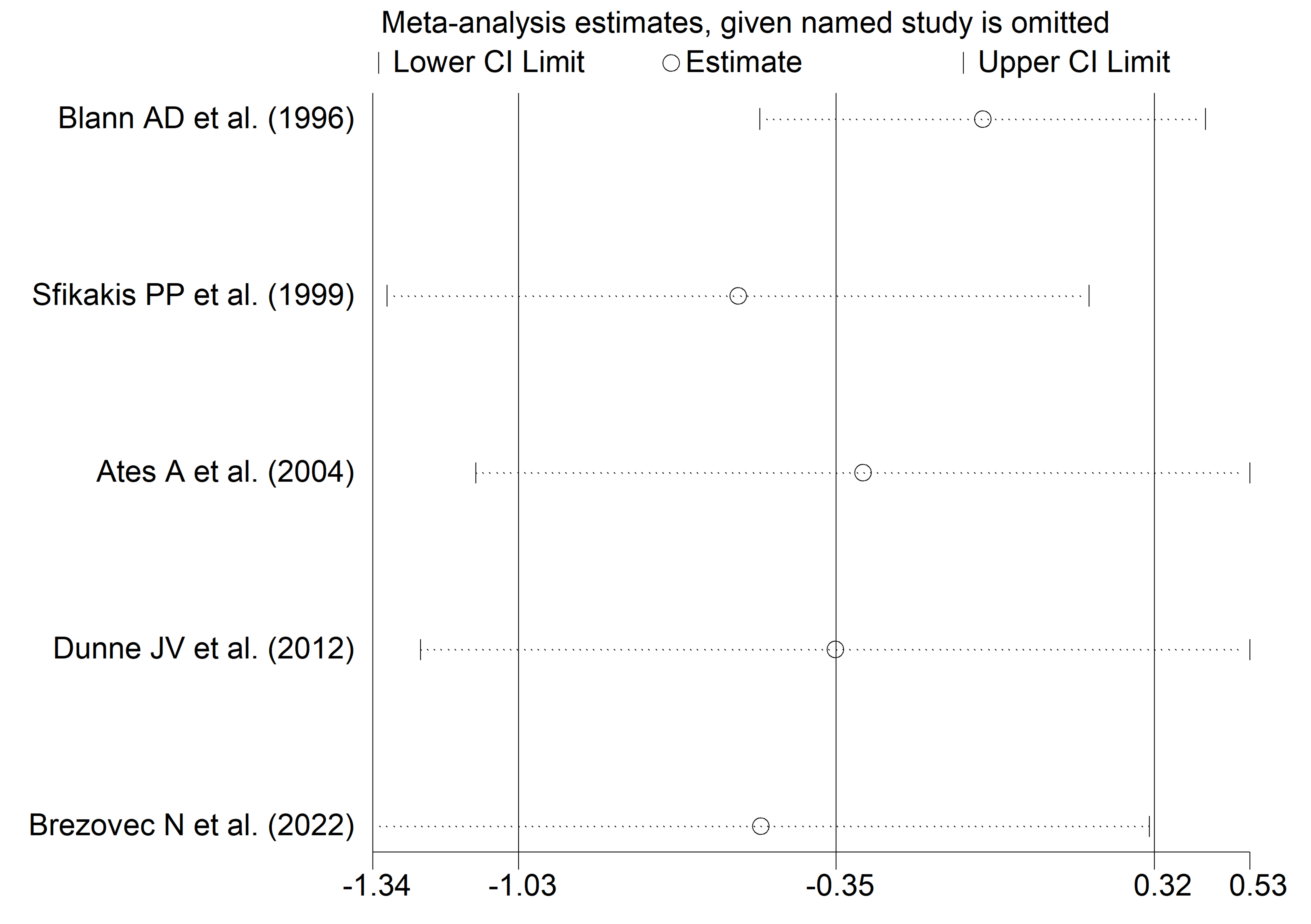

Supplement: Supplementary Figure 8 — Sensitivity analysis of the association between L-selectin concentrations and SSc. [file Image8.tif]

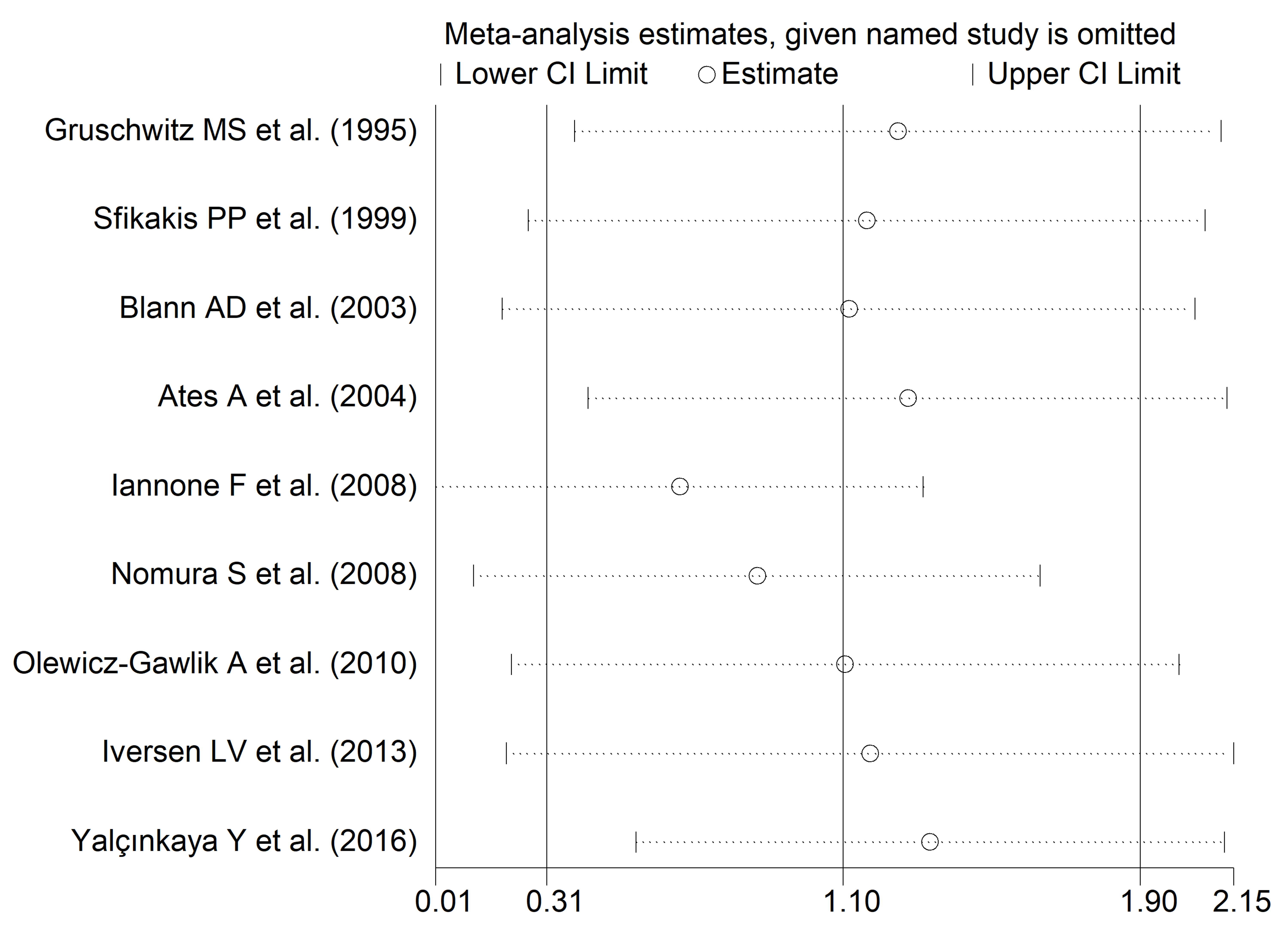

Supplement: Supplementary Figure 9 — Sensitivity analysis of the association between P-selectin concentrations and SSc. [file Image9.tif]
