# Supplementary material for: Circulating cell adhesion molecules in systemic sclerosis: a systematic review and meta-analysis
Source: Front Immunol. 2024 Aug 21;15:1438302. doi: 10.3389/fimmu.2024.1438302 (PMC11371573; doi:10.3389/fimmu.2024.1438302)
Supplement: Supplementary file 11 [file Table2.docx]

**Supplementary Table 2.** Assessment of the risk of bias.

| **Study** | **Were the inclusion criteria clearly defined?** | **Were the subjects and the setting described in detail?** | **Was the exposure measured in a reliable way?** | **Were standard criteria used to assess the condition?** | **Were confounding factors identified?** | **Were strategies to deal with confounding factors stated?** | **Were the outcomes measured in a reliable way?** | **Was appropriate statistical analysis used?** | **Risk of bias** |
| --- | --- | --- | --- | --- | --- | --- | --- | --- | --- |
| Carson CW et al. (1) | No | No | Yes | Yes | No | No | Yes | Yes | Moderate |
| Sfikakis PP et al. (2) | No | Yes | Yes | Yes | No | No | Yes | Yes | Moderate |
| Kiener K et al. (3) | No | Yes | Yes | Yes | No | No | Yes | Yes | Moderate |
| Blann AD et al. (4) | Yes | Yes | Yes | Yes | No | No | Yes | Yes | Low |
| Gruschwitz MS et al. (5) | Yes | Yes | Yes | Yes | No | No | Yes | Yes | Low |
| Blann AD et al. (6) | Yes | Yes | Yes | Yes | No | No | Yes | Yes | Low |
| Ihn H et al. (7) | No | Yes | Yes | Yes | No | No | Yes | Yes | Moderate |
| Ihn H et al. (8) | No | Yes | Yes | Yes | No | No | Yes | Yes | Moderate |
| Majewski S et al. (9) | No | Yes | Yes | Yes | No | No | Yes | Yes | Moderate |
| Sfikakis PP et al. (10) | Yes | Yes | Yes | Yes | No | No | Yes | Yes | Low |
| Andersen GN et al. (11) | No | Yes | Yes | Yes | Yes | Yes | Yes | Yes | Low |
| Macko RF et al. (12) | Yes | Yes | Yes | Yes | Yes | Yes | Yes | Yes | Low |
| Blann AD et al. (13) | Yes | Yes | Yes | Yes | No | No | Yes | Yes | Low |
| Cerinic MM et al. (14) | No | Yes | Yes | Yes | No | No | Yes | Yes | Moderate |
| Zamzam ML et al. (15) | No | Yes | Yes | Yes | No | No | Yes | Yes | Moderate |
| Allanore Y et al. (16) | Yes | Yes | Yes | Yes | No | No | Yes | Yes | Low |
| Ates A et al. (17) | Yes | Yes | Yes | Yes | No | No | Yes | Yes | Low |
| Kuryliszyn-Moskal A et al. (18) | No | Yes | Yes | Yes | No | No | Yes | Yes | Moderate |
| Dovio A et al. (19) | Yes | Yes | Yes | Yes | No | No | Yes | Yes | Low |
| Hettema ME et al. (20) | Yes | Yes | Yes | Yes | Yes | Yes | Yes | Yes | Low |
| Iannone F et al. (21) | Yes | Yes | Yes | Yes | No | No | Yes | Yes | Low |
| Nomura S et al. (22) | No | No | Yes | No | No | No | Yes | Yes | High |
| Minier T et al. (23) | No | Yes | Yes | Yes | No | No | Yes | Yes | Moderate |
| Olewicz-Gawlik A et al. (24) | No | Yes | Yes | Yes | No | No | Yes | Yes | Moderate |
| Alzawawy AI et al. (25) | Yes | Yes | Yes | Yes | No | No | Yes | Yes | Low |
| Riccieri V et al. (26) | No | Yes | Yes | Yes | No | No | Yes | Yes | Moderate |
| Dunne JV et al. (27) | Yes | Yes | Yes | Yes | No | No | Yes | Yes | Low |
| Aydoğdu E et al. (28) | Yes | Yes | Yes | Yes | No | No | Yes | Yes | Low |
| Iversen LV et al. (29) | Yes | Yes | Yes | Yes | No | No | Yes | Yes | Low |
| Cossu M et al. (30) | No | Yes | Yes | Yes | No | No | Yes | Yes | Moderate |
| Yalçınkaya Y et al. (31) | No | Yes | Yes | Yes | No | No | Yes | Yes | Moderate |
| Delle Sedie A et al. (32) | No | Yes | Yes | Yes | No | No | Yes | Yes | Moderate |
| Thakkar V et al. (33) | Yes | Yes | Yes | Yes | No | No | Yes | Yes | Low |
| Wodok-Wieczorek K et al. (34) | Yes | Yes | Yes | Yes | No | No | Yes | Yes | Low |
| Hegazy GA et al. (35) | Yes | Yes | Yes | Yes | No | No | Yes | Yes | Low |
| Pacholczak-Madej R et al. (36) | Yes | Yes | Yes | Yes | No | No | Yes | Yes | Low |
| Al-Omary Obadeh M et al. (37) | No | Yes | Yes | Yes | No | No | Yes | Yes | Moderate |
| Kuszmiersz P et al. (38) | Yes | Yes | Yes | Yes | Yes | Yes | Yes | Yes | Low |
| Stern EP et al. (39) | No | Yes | Yes | Yes | No | No | Yes | Yes | Moderate |
| Brezovec N et al. (40) | No | Yes | Yes | Yes | No | No | Yes | Yes | Moderate |
| Colic J et al. (41) | Yes | Yes | Yes | Yes | No | No | Yes | Yes | Low |
| Jee AS et al. (42) | No | Yes | Yes | Yes | Yes | Yes | Yes | Yes | Low |
| Corrado A et al. (43) | Yes | No | Yes | Yes | Yes | Yes | Yes | Yes | Low |

**References**

1. Carson CW, Beall LD, Hunder GG, Johnson CM, Newman W. Serum ELAM-1 is increased in vasculitis, scleroderma, and systemic lupus erythematosus. J Rheumatol. 1993;20(5):809-14. doi:

2. Sfikakis PP, Tesar J, Baraf H, Lipnick R, Klipple G, Tsokos GC. Circulating intercellular adhesion molecule-1 in patients with systemic sclerosis. Clin Immunol Immunopathol. 1993;68(1):88-92. doi: 10.1006/clin.1993.1100

3. Kiener H, Graninger W, Machold K, Aringer M, Graninger WB. Increased levels of circulating intercellular adhesion molecule-1 in patients with systemic sclerosis. Clin Exp Rheumatol. 1994;12(5):483-7. doi:

4. Blann AD, Herrick A, Jayson MI. Altered levels of soluble adhesion molecules in rheumatoid arthritis, vasculitis and systemic sclerosis. Br J Rheumatol. 1995;34(9):814-9. doi: 10.1093/rheumatology/34.9.814

5. Gruschwitz MS, Hornstein OP, von Den Driesch P. Correlation of soluble adhesion molecules in the peripheral blood of scleroderma patients with their in situ expression and with disease activity. Arthritis Rheum. 1995;38(2):184-9. doi: 10.1002/art.1780380206

6. Blann AD, Sanders PA, Herrick A, Jayson MI. Soluble L-selectin in the connective tissue diseases. Br J Haematol. 1996;95(1):192-4. doi: 10.1046/j.1365-2141.1996.7562378.x

7. Ihn H, Sato S, Fujimoto M, Kikuchi K, Kadono T, Tamaki K, et al. Circulating intercellular adhesion molecule-1 in the sera of patients with systemic sclerosis: enhancement by inflammatory cytokines. Br J Rheumatol. 1997;36(12):1270-5. doi: 10.1093/rheumatology/36.12.1270

8. Ihn H, Sato S, Fujimoto M, Takehara K, Tamaki K. Increased serum levels of soluble vascular cell adhesion molecule-1 and E-selectin in patients with systemic sclerosis. Br J Rheumatol. 1998;37(11):1188-92. doi: 10.1093/rheumatology/37.11.1188

9. Majewski S, Wojas-Pelc A, Malejczyk M, Szymanska E, Jablonska S. Serum levels of soluble TNF alpha receptor type I and the severity of systemic sclerosis. Acta Derm Venereol. 1999;79(3):207-10. doi: 10.1080/000155599750010986

10. Sfikakis PP, Charalambopoulos D, Vaiopoulos G, Mavrikakis M. Circulating P- and L-selectin and T-lymphocyte activation and patients with autoimmune rheumatic diseases. Clin Rheumatol. 1999;18(1):28-32. doi: 10.1007/s100670050047

11. Andersen GN, Mincheva‐Nilsson L, Kazzam E, Nyberg G, Klintland N, Petersson AS, et al. Assessment of vascular function in systemic sclerosis: Indications of the development of nitrate tolerance as a result of enhanced endothelial nitric oxide production. Arthritis & Rheumatism. 2002;46(5):1324-32. doi: 10.1002/art.10191

12. Macko RF, Gelber AC, Young BA, Lowitt MH, White B, Wigley FM, et al. Increased circulating concentrations of the counteradhesive proteins SPARC and thrombospondin-1 in systemic sclerosis (scleroderma). Relationship to platelet and endothelial cell activation. J Rheumatol. 2002;29(12):2565-70. doi:

13. Blann AD, Constans J, Carpentier P, Renard M, Satger B, Guérin V, et al. Soluble P selectin in systemic sclerosis: relationship with von Willebrand factor, autoantibodies and diffuse or localised/limited disease. Thrombosis Research. 2003;109(4):203-6. doi: 10.1016/s0049-3848(03)00209-3

14. Cerinic MM, Valentini G, Sorano GG, D'Angelo S, Cuomo G, Fenu L, et al. Blood coagulation, fibrinolysis, and markers of endothelial dysfunction in systemic sclerosis. Seminars in Arthritis and Rheumatism. 2003;32(5):285-95. doi: 10.1053/sarh.2002.50011

15. Zamzam ML, Yassin MM, Sallam MM. Implication of intercellular adhesion molecule-1 (ICAM-1) and serum N(G)-hydroxy-L-arginine (L-NHA) in the pathogenesis of systemic sclerosis. Egypt J Immunol. 2003;10(2):27-38. doi:

16. Allanore Y, Borderie D, Lemarechal H, Ekindjian OG, Kahan A. Nifedipine decreases sVCAM-1 concentrations and oxidative stress in systemic sclerosis but does not affect the concentrations of vascular endothelial growth factor or its soluble receptor 1. Arthritis Res Ther. 2004;6(4):R309-14. doi: 10.1186/ar1183

17. Ateş A, Kinikli G, Turgay M, Duman M. Serum‐Soluble Selectin Levels in Patients with Rheumatoid Arthritis and Systemic Sclerosis. Scandinavian Journal of Immunology. 2004;59(3):315-20. doi: 10.1111/j.0300-9475.2004.01389.x

18. Kuryliszyn-Moskal A, Klimiuk PA, Sierakowski S. Soluble adhesion molecules (sVCAM-1, sE-selectin), vascular endothelial growth factor (VEGF) and endothelin-1 in patients with systemic sclerosis: relationship to organ systemic involvement. Clinical Rheumatology. 2004;24(2):111-6. doi: 10.1007/s10067-004-0987-3

19. Dovio A, Data V, Carignola R, Calzolari G, Vitetta R, Ventura M, et al. Circulating Osteoprotegerin and Soluble RANK Ligand in Systemic Sclerosis. The Journal of Rheumatology. 2008;35(11):2206-13. doi: 10.3899/jrheum.080192

20. Hettema ME, Zhang D, de Leeuw K, Stienstra Y, Smit AJ, Kallenberg CGM, et al. Early atherosclerosis in systemic sclerosis and its relation to disease or traditional risk factors. Arthritis Research & Therapy. 2008;10(2). doi: 10.1186/ar2408

21. Iannone F, Riccardi MT, Guiducci S, Bizzoca R, Cinelli M, Matucci-Cerinic M, et al. Bosentan regulates the expression of adhesion molecules on circulating T cells and serum soluble adhesion molecules in systemic sclerosis-associated pulmonary arterial hypertension. Annals of the Rheumatic Diseases. 2008;67(8):1121-6. doi: 10.1136/ard.2007.080424

22. Nomura S, Inami N, Ozaki Y, Kagawa H, Fukuhara S. Significance of microparticles in progressive systemic sclerosis with interstitial pneumonia. Platelets. 2009;19(3):192-8. doi: 10.1080/09537100701882038

23. Minier T, Nagy Z, Balint Z, Farkas H, Radics J, Kumanovics G, et al. Construct validity evaluation of the European Scleroderma Study Group activity index, and investigation of possible new disease activity markers in systemic sclerosis. Rheumatology. 2010;49(6):1133-45. doi: 10.1093/rheumatology/keq022

24. Olewicz-Gawlik A, Danczak-Pazdrowska A, Klama K, Silny W, Prokop J, Mackiewicz S, et al. Blood Serum Levels of Amino-Terminal Pro-C-Type Natriuretic Peptide in Patients with Systemic Sclerosis. Connective Tissue Research. 2010;51(2):83-7. doi: 10.3109/03008200903056168

25. Alzawawy AI, Suliman I, Hamimi A, Elsawy N, Albordiny MM. Serum soluble vascular cell adhesion molecule-1 (sVCAM-1) in scleroderma patients and its relation to pulmonary involvement and disease activity. The Egyptian Rheumatologist. 2011;33(1):21-6. doi: 10.1016/j.ejr.2010.06.001

26. Riccieri V, Stefanantoni K, Vasile M, Macri V, Sciarra I, Iannace N, et al. Abnormal plasma levels of different angiogenic molecules are associated with different clinical manifestations in patients with systemic sclerosis. Clin Exp Rheumatol. 2011;29(2 Suppl 65):S46-52. doi:

27. Shi S, Dunne JV, van Eeden SF, Keen KJ. L-selectin and Skin Damage in Systemic Sclerosis. PLoS ONE. 2012;7(9). doi: 10.1371/journal.pone.0044814

28. Aydoğdu E, Pamuk ÖN, Dönmez S, Pamuk GE. Decreased interleukin-20 level in patients with systemic sclerosis: are they related with angiogenesis? Clinical Rheumatology. 2013;32(11):1599-603. doi: 10.1007/s10067-013-2317-0

29. Iversen LV, Østergaard O, Ullman S, Nielsen CT, Halberg P, Karlsmark T, et al. Circulating microparticles and plasma levels of soluble E- and P-selectins in patients with systemic sclerosis. Scandinavian Journal of Rheumatology. 2013;42(6):473-82. doi: 10.3109/03009742.2013.796403

30. Cossu M, Andracco R, Santaniello A, Marchini M, Severino A, Caronni M, et al. Serum levels of vascular dysfunction markers reflect disease severity and stage in systemic sclerosis patients. Rheumatology. 2016;55(6):1112-6. doi: 10.1093/rheumatology/kew017

31. Yalçınkaya Y, Adın- Çınar S, Artim-Esen B, Kamalı S, Pehlivan Ö, Öcal L, et al. Capillaroscopic findings and vascular biomarkers in systemic sclerosis: Association of low CD40L levels with late scleroderma pattern. Microvascular Research. 2016;108:17-21. doi: 10.1016/j.mvr.2016.07.002

32. Delle Sedie A, Riente L, Maggiorini L, Pratesi F, Tavoni A, Migliorini P, et al. Potential biomarkers in patients with systemic sclerosis. International Journal of Rheumatic Diseases. 2018;21(1):261-5. doi: 10.1111/1756-185x.13196

33. Thakkar V, Patterson KA, Stevens W, Wilson M, Roddy J, Sahhar J, et al. Increased serum levels of adhesion molecules ICAM-1 and VCAM-1 in systemic sclerosis are not specific for pulmonary manifestations. Clinical Rheumatology. 2018;37(6):1563-71. doi: 10.1007/s10067-018-4081-7

34. Wodok-Wieczorek K, Salwowska N, Syguła E, Wodok A, Wcisło-Dziadecka D, Bebenek K, et al. The correlation between serum E-selectin levels and soluble interleukin-2 receptors with relation to disease activity in localized scleroderma. Advances in Dermatology and Allergology. 2018;35(6):614-9. doi: 10.5114/ada.2018.77613

35. A. Hegazy G, Shaker O, Sayed S, Elzaher AA, Fathy K, Wahby I, et al. Biomarkers of Systemic Lupus Erythematosus and Systemic Sclerosis diseases activity in a sample of Egyptian patients :Soluble Intercellular Adhesion Molecule-1 and Soluble Interleukin-2 Receptor, Case Control Study. Biomedical & Pharmacology Journal. 2019;12(3):1207-16. doi: 10.13005/bpj/1750

36. Pacholczak-Madej R, Kuszmiersz P, Bazan-Socha S, Kosałka-Węgiel J, Iwaniec T, Zaręba L, et al. Endothelial dysfunction in patients with systemic sclerosis. Advances in Dermatology and Allergology. 2020;37(4):495-502. doi: 10.5114/ada.2019.83501

37. Al-Omary Obadeh M, Bondar S. Endothelial Dysfunction and Pathogenetic Phenotypes of Localized Scleroderma. Georgian Med News. 2021(319):102-8. doi:

38. Kuszmiersz P, Pacholczak-Madej R, Siwiec A, Celinska-Lowenhoff M, Iwaniec T, Kosalka-Wegiel J, et al. Thrombin generation potential is enhanced in systemic sclerosis: impact of selected endothelial biomarkers. Clin Exp Rheumatol. 2021;39 Suppl 131(4):13-9. doi: 10.55563/clinexprheumatol/d03dnc

39. Stern EP, Unwin R, Burns A, Ong VH, Denton CP. Exploring molecular pathology of chronic kidney disease in systemic sclerosis by analysis of urinary and serum proteins. Rheumatology Advances in Practice. 2021;5(1). doi: 10.1093/rap/rkaa083

40. Brezovec N, Perdan-Pirkmajer K, Kuret T, Burja B, Sodin-Šemrl S, Čučnik S, et al. Increased L-Selectin on Monocytes Is Linked to the Autoantibody Profile in Systemic Sclerosis. International Journal of Molecular Sciences. 2022;23(4). doi: 10.3390/ijms23042233

41. Colic J, Pruner I, Damjanov N, Pekmezovic T, Sefik-Bukilica M, Antovic A. Impaired Fibrinolysis Is Linked With Digital Vasculopathy and Onset of New Digital Ulcers in Systemic Sclerosis. The Journal of Rheumatology. 2022;49(6):598-606. doi: 10.3899/jrheum.210931

42. Jee AS, Stewart I, Youssef P, Adelstein S, Lai D, Hua S, et al. A Composite Serum Biomarker Index for the Diagnosis of Systemic Sclerosis-Associated Interstitial Lung Disease: A Multicenter, Observational Cohort Study. Arthritis Rheumatol. 2023;75(8):1424-33. doi: 10.1002/art.42491

43. Corrado A, Mansueto N, Correale M, Rella V, Tricarico L, Altomare A, et al. Flow Mediated Dilation in Systemic Sclerosis: Association with clinical findings, capillaroscopic patterns and endothelial circulating markers. Vascular Pharmacology. 2024;154. doi: 10.1016/j.vph.2023.107252
